# Targeting GM-CSF in Rheumatoid Arthritis: Advances in Cytokine-Directed Immunotherapy and Clinical Implications

**DOI:** 10.3390/life15111737

**Published:** 2025-11-12

**Authors:** Mario García-Domínguez

**Affiliations:** 1 Program of Immunology and Immunotherapy, CIMA-Universidad de Navarra, 31008 Pamplona, Spain; mgdom@unav.es; 2 Department of Immunology and Immunotherapy, Clínica Universidad de Navarra, 31008 Pamplona, Spain; 3 Centro de Investigación Biomédica en Red de Cáncer (CIBERONC), 28029 Madrid, Spain

**Keywords:** rheumatoid arthritis, GM-CSF, inflammation, synovial inflammation, pro-inflammatory cytokines, synovitis, immunomodulation, autoimmunity

## Abstract

Granulocyte-macrophage colony-stimulating factor (GM-CSF) has emerged as a key cytokine in the pathogenesis of rheumatoid arthritis, an autoimmune disease distinguished by synovial inflammation and progressive joint destruction. GM-CSF orchestrates the activation, proliferation, and differentiation of myeloid cells (mainly macrophages and neutrophils) thereby sustaining the pro-inflammatory synovial milieu. Recent advances in monoclonal antibody immunotherapy have enabled selective inhibition of GM-CSF or its receptor. Clinical data on several monoclonal antibodies are presented, focusing on their pharmacodynamic properties and efficacy results documented in phase II and III clinical studies. Cumulative evidence supports GM-CSF inhibition as a compelling strategy for modulating inflammation and improving clinical outcomes in rheumatoid arthritis.

## 1. Introduction

Rheumatoid arthritis (RA) is a chronic, systemic autoimmune disorder characterized by persistent synovial inflammation, pannus formation, and destruction of articular cartilage and subchondral bone [1]. This disease arises from a multifactorial interplay of genetic risk factors, environmental triggers, and dysregulated immune responses that culminate in chronic joint inflammation and systemic comorbidities [2,3]. RA pathogenesis involves the aberrant activation of both innate and adaptive immune compartments, including macrophages, neutrophils, dendritic cells, Th1 and Th17 lymphocytes, and autoreactive B cells producing rheumatoid factor (RF) and anti-citrullinated protein antibodies (ACPAs) [4,5,6]. These immune populations generate a pathogenic cytokine milieu dominated by TNF-α, IL-1β, IL-6, IL-17, and granulocyte-macrophage colony-stimulating factor (GM-CSF), which sustains synovial inflammation and drives structural joint damage [7,8,9].

Among these mediators, GM-CSF has gained increasing attention as a central regulator of myeloid cell activation within the inflamed synovium [10,11]. GM-CSF is produced by activated T cells, fibroblast-like synoviocytes (FLS), endothelial cells, and macrophages in response to pro-inflammatory cues such as TNF-α and IL-1β [12,13]. Its effects are mediated through binding to the heterodimeric GM-CSF receptor (GM-CSFR), composed of a cytokine-specific α-chain (GM-CSFRα) and a signal-transducing common β-chain (GM-CSFRβc), functionally shared with the IL-3 and IL-5 receptor families [14,15]. Ligand engagement induces receptor dimerization and activates downstream signaling cascades, mainly JAK2-STAT5, MAPK/ERK, PI3K/Akt, and NF-κB pathways, resulting in enhanced survival, differentiation, and effector function of myeloid cells [16,17,18,19].

In RA, dysregulated GM-CSF signaling elicits the differentiation of circulating monocytes into pro-inflammatory macrophages that secrete TNF-α, IL-6, and IL-23, reinforcing a pathogenic axis with Th17 cells [20,21]. GM-CSF further augments neutrophil survival, oxidative burst, and degranulation, and enhances dendritic cell maturation and antigen presentation, thereby sustaining autoreactive T-cell activation and chronic synovitis [15,22]. These mechanisms collectively amplify a self-reinforcing inflammatory circuit that accelerates osteoclastogenesis, cartilage degradation, and bone erosion [22].

Despite the substantial therapeutic progress achieved with conventional and biologic disease-modifying antirheumatic drugs (DMARDs) have transformed RA management, 30–40% of patients display inadequate clinical responses or develop secondary resistance [23,24,25]. This therapeutic limitation has prompted investigation of alternative cytokine targets upstream of established inflammatory cascades. Blockade of the GM-CSF pathway offers a mechanistically rational strategy to interfere with early myeloid-driven inflammation. Neutralizing antibodies directed against GM-CSF (e.g., otilimab and namilumab) or GM-CSFRα (e.g., mavrilimumab) facilitate selective interruption of this signaling axis [26,27]. Preclinical models demonstrate that GM-CSF inhibition reduces synovial macrophage accumulation, pro-inflammatory cytokine production, and osteoclast differentiation, thereby preventing structural joint damage [15,28].

Translation of these findings into clinical settings has been supported by Phase II and III trials evaluating mavrilimumab, otilimab, and namilumab, which have shown significant improvements in some disease activity indices (e.g., DAS28 and ACR responses) and patient-reported outcomes, accompanied by favorable safety profiles [29,30]. Mild respiratory adverse events observed in some studies reflect the physiological role of GM-CSF in pulmonary macrophage homeostasis, underscoring the importance of balanced cytokine modulation [31]. Comparative analyses suggest that GM-CSF blockade might provide additive or complementary benefit, particularly in patients with myeloid-dominant disease or insufficient response to TNF-α inhibition.

This review synthesizes current mechanistic insights and clinical evidence regarding GM-CSF and GM-CSFR inhibition in RA, with the aim of critically assessing the therapeutic potential of targeting this pathway as an innovative strategy for durable immune modulation and improved disease control. Additionally, this review addresses the knowledge gap concerning the translational relevance of GM-CSF blockade, highlighting novel perspectives that have not yet been integrated into the current literature, thereby underscoring its potential to reshape future therapeutic paradigms in RA.

## 2. Fundamental Aspects of RA

RA is a chronic, systemic, autoimmune disease characterized by persistent synovial inflammation, progressive joint destruction, and many extra-articular manifestations [1]. It represents one of the most prevalent autoimmune disorders worldwide, affecting approximately 0.5 to 1% of the adult population [32], exhibiting a marked female predominance (3:1) with peak incidence occurring between 40 and 60 years [33]. The disease course is typically progressive and fluctuating, with periods of exacerbation and remission [34]. If inadequately treated, it can cause considerable functional impairment, functional disability, and premature mortality, predominantly attributable to cardiovascular complications and systemic inflammation [35,36].

The etiology of RA is multifactorial, involving a complex interplay of genetic, environmental, hormonal, and immunological factors [2]. Genetic susceptibility accounts for nearly half of the risk, and among genetic determinants, the strongest association has been established with alleles of the *HLA-DRB1* gene encoding the so-called “shared epitope”, which influences antigen presentation to CD4^+^ T lymphocytes [37,38]. Other genetic loci, such as *PTPN22*, *STAT4*, and *CTLA4*, contribute to immune dysregulation and loss of tolerance [39,40,41]. Environmental factors, especially cigarette smoking, have been identified as major triggers in genetically predisposed individuals, enhancing protein citrullination and the generation of neoantigens that stimulate autoimmunity [42]. Additional environmental and microbial agents, including *Porphyromonas gingivalis* and *Aggregatibacter actinomycetemcomitans*, have been implicated in the pathogenesis through similar mechanisms involving post-translational protein modifications [43,44]. Hormonal influences, like estrogen fluctuations and pregnancy-associated immune modulation, might further modify disease risk and activity [45].

**Figure 1:**
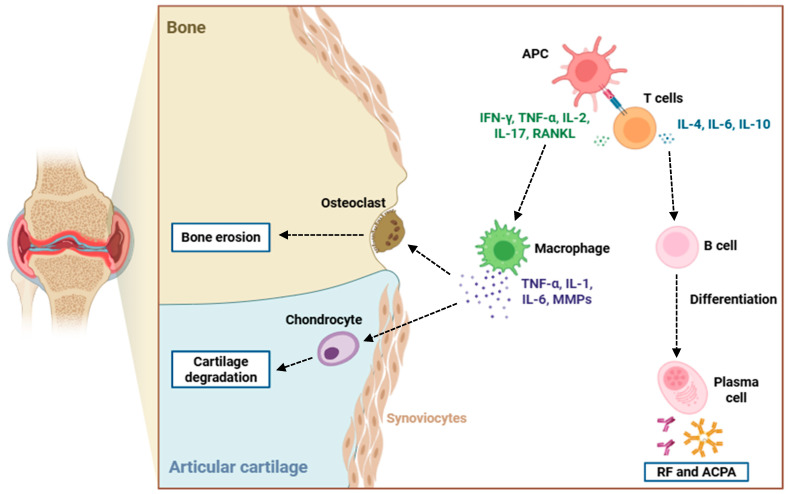
Immunopathogenesis of bone and cartilage destruction in RA. This figure illustrates the cellular and molecular mechanisms driving joint damage in RA. APCs activate T cells, which release pro-inflammatory cytokines like IFN-γ, TNF-α, IL-2, IL-17, and RANKL, promoting macrophage activation and osteoclast differentiation. Activated macrophages secrete and release TNF-α, IL-1, IL-6, and MMPs, contributing to synovial inflammation, chondrocyte-mediated cartilage degradation, and osteoclast-induced bone erosion. Concurrently, T cells stimulate B cell differentiation into plasma cells that produce RF and ACPAs, further amplifying the autoimmune response. The interplay among these immune cells and cytokines leads to the progressive destruction of articular cartilage and bone typical of RA pathology. Abbreviations: APC (antigen-presenting cell), RF (rheumatoid factor), ACPA (anti-citrullinated protein antibody), IFN-γ (interferon-gamma), TNF-α (tumor necrosis factor-alpha), IL-1 (interleukin 1), IL-2 (interleukin 2), IL-4 (interleukin 4), IL-6 (interleukin 6), IL-10 (interleukin 10), IL-17 (interleukin 17), RANKL (receptor activator of nuclear factor kappa-B ligand), and MMPs (matrix metalloproteinases).

The immunopathogenesis of RA is characterized by a loss of self-tolerance, resulting in the activation of autoreactive T and B lymphocytes (Figure 1) [46]. Autoreactive CD4^+^ T cells, including Th1 and Th17 subsets, infiltrate the synovial tissue, where they interact with local antigen-presenting cells (APCs) like dendritic cells and macrophages [47,48]. Th1 cells secrete IFN-γ, enhancing macrophage activation, while Th17 cells produce IL-17A, IL-17F, and IL-22, which synergize with TNF-α, IL-1β, and IL-6 to amplify inflammation and recruit neutrophils and monocytes to the synovium [49]. Regulatory T cell (Treg) dysfunction in RA further exacerbates this pro-inflammatory milieu, resulting in uncontrolled effector T cell activity [50]. Under these conditions, FLS adopt a pathogenic, hyperactivated phenotype, marked by increased proliferative capacity and resistance to programmed cell death, and increased production of matrix metalloproteinases (MMPs), such as MMP-1, MMP-3, and MMP-9, which mediate degradation of the cartilage extracellular matrix [51,52]. FLS also secrete chemokines such as CCL2, CCL5, and CXCL10, recruiting additional immune cells and sustaining local inflammation [53].

On the other hand, B cells contribute to RA pathogenesis via many mechanisms. They differentiate into plasma cells that secrete autoantibodies, most prominently RF and ACPAs [54]. These autoantibodies induce the formation of immune complexes that activate the classical complement pathway, generating C3a and C5a fragments that act as chemoattractants for neutrophils and monocytes, further amplifying synovial inflammation [55]. B cells also function as antigen-presenting cells, stimulating autoreactive T cells, and secrete pro-inflammatory cytokines like IL-6, TNF-α, and lymphotoxin, thereby perpetuating local immune activation [56,57].

Neutrophils contribute through the release of reactive oxygen species (ROS) and neutrophil extracellular traps (NETs), which not only damage synovial tissue but also provide citrullinated antigens that fuel ACPA production [58]. Monocytes and macrophages in the synovium adopt a pro-inflammatory M1 phenotype (Figure 1), secreting TNF-α, IL-1β, IL-6, and IL-12, which promote Th1/Th17 responses and enhance osteoclastogenesis [59].

Chronic inflammation drives synovial hyperplasia and the formation of pannus tissue, an invasive structure that erodes articular cartilage and subchondral bone [60]. Osteoclast differentiation is mainly mediated by receptor activator of nuclear factor κB ligand (RANKL), located at activated T cells, FLS, and synovial macrophages [61]. RANKL binds to its receptor RANK on osteoclast precursors, triggering NF-κB, NFATc1, and c-Fos signaling pathways, which culminate in osteoclast maturation and activation [62]. Other contributing modulators of osteoclastogenesis include TNF-α, IL-17, and M-CSF, further enhance bone resorption [63].

Clinically, RA typically presents as a symmetrical polyarthritis mainly involving the joints of the hands (metacarpophalangeal and proximal interphalangeal), wrists, and feet (metatarsophalangeal joints) [64,65]. The onset is typically insidious, characterized by articular pain, swelling, increased warmth, and prolonged morning stiffness persisting for more than one hour [66]. Fatigue, low-grade fever, and malaise are common symptoms [67]. As the disease advances, chronic synovitis leads to joint deformities including ulnar deviation, boutonnière and swan-neck deformities, and subluxations, leading to progressive loss of function [68]. Extra-articular manifestations occur in approximately 30–40% of patients and may include rheumatoid nodules, vasculitis, pleural and pericardial effusions, interstitial lung disease, peripheral neuropathy, scleritis, and hematological abnormalities (e.g., anemia and thrombocytosis) [69]. Cardiovascular involvement, particularly accelerated atherosclerosis, contributes significantly to the increased mortality observed in RA populations [70].

Finally, diagnosis is based on the synthesis of clinical assessment, serological testing, and imaging modalities [71]. The 2010 ACR/EULAR criteria integrate the number and distribution of affected joints, serological markers (e.g., RF and ACPA), acute-phase reactants such as C-reactive protein (CRP) and erythrocyte sedimentation rate (ESR), and symptom duration [72]. ACPA testing, mainly anti-cyclic citrullinated peptide (anti-CCP) antibodies, provides high specificity for RA and may precede clinical onset by several years [73]. Imaging modalities, including ultrasonography and magnetic resonance imaging (MRI), facilitate the detection of early synovitis, tenosynovitis, and erosions that are not apparent on conventional radiographs, thus facilitating early diagnosis [74].

## 3. Role of GM-CSF in RA

GM-CSF is a pleiotropic cytokine that plays a central role in the immunopathogenesis of RA. Its multifaceted actions on myeloid cell differentiation, activation, survival, and effector function make GM-CSF a critical driver of chronic synovial inflammation, pannus formation, and joint destruction [10,11]. In RA, GM-CSF acts not merely as a hematopoietic growth factor but also as a key pro-inflammatory mediator coordinating innate and adaptive immune responses. This section provides an in-depth exploration of the molecular and cellular mechanisms by which GM-CSF contributes to RA pathogenesis.

### 3.1. Molecular Signaling Mechanisms of GM-CSF

GM-CSF mediates its diverse biological effects mainly through GM-CSFR, a heterodimeric transmembrane complex composed of a ligand-specific α-chain (GM-CSFRα) and a common βc signaling subunit (GM-CSFRβc) shared with the IL-3 and IL-5 receptors [75]. The α-chain is a single-pass transmembrane protein that mediates ligand recognition [76]. Its extracellular domain comprises modular motifs typical of type I cytokine receptors, including a cytokine receptor homology domain (CHD) with conserved fibronectin type III-like subdomains. Although the α-chain lacks intrinsic signaling capability, it serves a structural function by capturing GM-CSF and presenting it in a conformation that facilitates βc subunit engagement [77]. On the other hand, the βc subunit functions as a multifunctional signaling component that mediates receptor dimerization, stability, and downstream signaling [78]. Its extracellular domain facilitates low-affinity ligand binding and heterodimerization with GM-CSFRα through fibronectin type III-like repeats that stabilize the receptor-ligand complex [79]. The transmembrane region promotes receptor oligomerization through helix-helix interactions, promoting the spatial alignment of cytoplasmic domains. The cytoplasmic tail, enriched in conserved tyrosine residues, provides docking sites for signaling molecules [80]. Ligand binding induces conformational rearrangements that bring βc cytoplasmic domains together, enabling recruitment and activation of JAK2, which triggers subsequent intracellular signaling cascades [81].

Activated JAK2 phosphorylates multiple conserved tyrosine residues within the cytoplasmic tail of the βc subunit, creating docking sites for several STAT proteins, mainly STAT5 [82]. STAT5 is subsequently phosphorylated, dimerize, and translocate to the nucleus, where they bind to promoter regions of some target genes to regulate transcription of critical effectors controlling cell survival (e.g., BCL2, MCL1, and BCL-XL), proliferation (e.g., cyclin D1 and c-Myc), and pro-inflammatory cytokine production (e.g., TNF-α, IL-1β, IL-6, and IL-23) [83,84,85]. This STAT5-dependent transcriptional program is critical for promoting the differentiation of monocytes into pro-inflammatory macrophages and dendritic cells within the synovial microenvironment [86].

Parallel to the JAK/STAT axis, GM-CSFR engagement activates PI3K/Akt signaling [87]. PI3K binds phosphorylated tyrosine residues on the receptor via its SH2 domains, catalyzing the conversion of PIP2 to PIP3 and recruiting Akt to the plasma membrane [88]. Akt undergoes phosphorylation by PDK1 and mTORC2, commencing a signaling cascade that promotes metabolic reprogramming, glycolytic flux, mitochondrial biogenesis, and inhibition of apoptotic pathways [89,90]. In parallel, GM-CSF receptor engagement stimulates the Ras/Raf/MEK/ERK (MAPK) pathway [91]. Upon activation, Ras engages Raf-1 kinase, promoting MEK1/2 phosphorylation and thus triggering ERK1/2 activation [92]. Nuclear translocation of ERK1/2 promotes phosphorylation of transcription factors such as Elk-1 and AP-1, driving the expression of inflammatory mediators (e.g., MMP-1, MMP-3, and MMP-13) and chemokines (CCL2, CCL3, and CXCL8) that orchestrate immune cell recruitment and synovial tissue remodeling [93,94,95].

Finally, GM-CSFR signaling activates the NF-κB pathway via phosphorylation and degradation of IκBα through the IKK complex [96]. Upon release, NF-κB translocates to the nucleus and drives expression of genes for pro-inflammatory cytokines, adhesion molecules, and survival factors, sustaining chronic inflammation [97]. Crosstalk between the JAK/STAT, PI3K/Akt, MAPK, and NF-κB pathways strengthens the transcriptional output, resulting in synergistic effects on myeloid cell activation, cytokine secretion, and tissue-destructive capacity.

### 3.2. Impact of GM-CSF on Immune Effector Cell Differentiation and Activation

**Figure 2:**
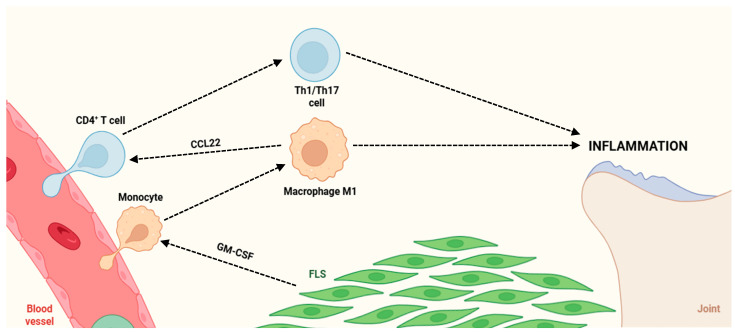
Pathogenic crosstalk between immune cells and FLS drives chronic joint inflammation. Circulating monocytes extravasate from the blood vessel into the synovial tissue, where GM-CSF produced by FLS promotes their differentiation into pro-inflammatory M1 macrophages. Activated M1 macrophages secrete the chemokine CCL22, which recruits CD4^+^ T cells and promotes their differentiation into Th1/Th17 effector subsets, establishing a self-amplifying inflammatory loop which sustains chronic joint inflammation. Abbreviations: CD4 (cluster of differentiation 4), Th1 (T helper 1 cell), Th17 (T helper 17 cell), CCL22 (chemokine (C-C motif) ligand 22), FLS (fibroblast-like synoviocytes), and GM-CSF (granulocyte-macrophage colony-stimulating factor).

In RA synovium, GM-CSF is synthesized by numerous resident and infiltrating cells (Figure 2), including FLS, Th17 lymphocytes, B cells, and endothelial cells, functioning via autocrine and paracrine signaling pathways to perpetuate synovial inflammation [98]. GM-CSF induces FLS to secrete IL-6, IL-8, and MMPs, which amplify leukocyte recruitment, promote angiogenesis, and drive cartilage degradation [99,100]. Molecularly, this involves activation of the GM-CSFR/JAK2/STAT5 axis in FLS, converging on NF-κB and AP-1 transcription factors, leading to increased expression of several pro-inflammatory mediators [101].

On the other hand, GM-CSF exerts pleiotropic effects on cells of the myeloid lineage (e.g., monocytes, macrophages, dendritic cells, and neutrophils) all of which play central roles in the pathogenesis of RA [102]. In macrophages, GM-CSF drives macrophage polarization toward a pro-inflammatory M1-like phenotype, marked by inducible nitric oxide synthase (iNOS) upregulation and augmented NO production, contributing to microbial killing and oxidative tissue stress [103]. Simultaneously, GM-CSF primes monocytes and macrophages to promote costimulatory molecules (CD40 and CD86) and chemokine receptors (CCR2 and CX3CR1), enhancing chemotactic responsiveness and sustaining immune cell retention within the synovium [104]. Moreover, GM-CSF augments ROS production, thus intensifying the inflammatory microenvironment and exacerbating oxidative injury to adjacent tissues [105].

GM-CSF-differentiated macrophages (GM-DMs) in the RA synovium secrete several chemokines (e.g., CCL22), which attract CD4^+^ T cells into the inflamed tissue (Figure 2). Upon recruitment, these T cells differentiate into Th1 and Th17 subsets, driven by the cytokine milieu enriched in IL-1β, IL-6, and IL-23 [20,106]. The interaction between GM-DMs and T cells creates a positive feedback loop that amplifies synovial inflammation and joint destruction [107]. Notably, GM-CSF upregulates CCL22 expression in macrophages through activation of the transcription factor IRF4, underscoring a pivotal axis in the pathogenesis of RA [20].

In addition to these functions, GM-CSF stimulates the secretion of several matrix metalloproteinases (MMP-1, MMP-9, and MMP-13), proteolytic enzymes that degrade critical components of the extracellular matrix, such as collagen and proteoglycans, thus driving cartilage erosion and joint destruction in pathological contexts [108]. At the transcriptional level, GM-CSF potentiates this phenotype through activation of IRF5 and NF-κB signaling pathways, which direct the expression of some pro-inflammatory cytokines, chemokines, and effector molecules, establishing a self-propagating inflammatory state within the synovial microenvironment [109,110].

In dendritic cells, GM-CSF enhances the antigen-presenting function by promoting upregulation of major histocompatibility complex (MHC) class II molecules as well as co-stimulatory receptors including CD80 and CD86, thereby increasing the capacity of dendritic cells to activate naïve and autoreactive T cells [111,112]. GM-CSF-stimulated dendritic cells produce elevated levels of pro-inflammatory cytokines including IL-1β, IL-6, and IL-23, which are critical drivers of Th17 cell differentiation, expansion, and functional polarization [113]. Simultaneously, these dendritic cells increase IL-12 production, supporting Th1 lineage commitment and reinforcing a multi-faceted Th cell-mediated inflammatory response [114]. This finding establishes a self-amplifying loop in which GM-CSF-producing Th17 cells further stimulate myeloid cells, thus exacerbating synovial inflammation and maintaining tissue damage [115]. The reciprocal activation between myeloid cells and autoreactive T cells functionally couples innate and adaptive immune responses, orchestrating the persistence of chronic inflammatory processes.

Neutrophils exposed to GM-CSF show delayed apoptosis, prolonging their lifespan within inflammatory sites, while concurrently exhibiting enhanced degranulation, releasing proteases and antimicrobial peptides that contribute to tissue damage [116]. GM-CSF also promotes formation of neutrophil extracellular traps (NETs), a process dependent in part on PI3K/Akt-mediated stabilization of the anti-apoptotic protein MCL1, as well as activation of ERK1/2 and p38 MAPK signaling cascades [117,118]. These NETs often contain citrullinated proteins, which function as potent autoantigens capable of breaking immune tolerance and driving the autoimmune response, a key pathophysiological process of RA [119].

Finally, GM-CSF signaling converges with various metabolic reprogramming pathways in immune cells, notably macrophages and dendritic cells. Engagement of the GM-CSFR triggers downstream activation of the JAK2/STAT5 and PI3K/Akt/mTOR signaling cascades, resulting in the transcriptional upregulation of critical metabolic enzymes [120]. This signaling promotes enhanced glycolysis through upregulation of hexokinase 2 (HK2) and phosphofructokinase-1 (PFK1), as well as increased glutaminolysis through induction of glutaminase (GLS), thus providing biosynthetic precursors and energy to support sustained pro-inflammatory cytokine production, such as TNF-α, IL-6, and IL-1β [121,122].

## 4. Therapeutic Targeting of GM-CSF

### 4.1. Overview of Monoclonal Antibody Strategies: GM-CSF Neutralization vs. Receptor Blockade

The GM-CSF signaling axis has emerged as a central mediator in the pathophysiology of RA, integrating both innate and adaptive immune mechanisms to drive synovial inflammation, tissue destruction, and pain sensitization. Its pleiotropic activity on myeloid lineage cells, particularly monocytes, macrophages, and neutrophils, positions GM-CSF as a key amplifier of pro-inflammatory cytokine networks, such as TNF-α, IL-1β, and IL-6, while simultaneously enhancing antigen presentation and osteoclast differentiation [102]. Consequently, therapeutic strategies that interfere with GM-CSF signaling have become a major focus in the development of novel biologic disease-modifying antirheumatic drugs (bDMARDs) [123].

Two primary monoclonal antibody (mAb) strategies have been developed to target the GM-CSF pathway in RA: ligand neutralization and receptor blockade [124]. Ligand neutralization employs antibodies directed against the soluble GM-CSF molecule itself, effectively sequestering this cytokine and preventing it from binding to its receptor on several target cells [125]. This approach is exemplified by agents like otilimab and namilumab, which demonstrate high-affinity binding to GM-CSF and inhibit its interaction with GM-CSFRα. Ligand neutralization might also modulate systemic GM-CSF-dependent immune functions, including hematopoiesis and myeloid cell survival [126,127].

Receptor blockade, in contrast, targets the GM-CSF receptor itself, usually via mAbs directed against GM-CSFRα [128]. Mavrilimumab represents a prototypical agent of this approach, targeting GM-CSFRα on immune innate cells (monocytes, macrophages, and neutrophils) to block GM-CSF interaction and abrogate ensuing receptor-dependent signaling cascades. Receptor blockade offers theoretical advantages in mechanistic specificity by selectively acting on GM-CSFRα-expressing cells, thereby mitigating off-target systemic effects and enabling more localized modulation of myeloid activation within inflamed tissues [129]. Moreover, this strategy may ensure prolonged suppression of GM-CSF signaling, as it prevents both locally synthesized and circulating GM-CSF from triggering downstream receptor-dependent pathways [129].

Both strategies share the common objective of interrupting the pathological GM-CSF axis to reduce synovial inflammation and tissue damage, but their mechanistic distinctions have important implications for pharmacokinetics, pharmacodynamics, and clinical application. Ligand-neutralizing antibodies necessitate plasma concentrations to bind circulating GM-CSF and achieve ligand sequestration, whereas receptor-blocking antibodies depend on receptor occupancy and target-cell distribution, factors that might influence dosing strategies and the onset of clinical efficacy [130]. Numerous preclinical models suggest that receptor blockade may provide a faster and more potent inhibition of local inflammatory responses, while ligand neutralization may offer broader systemic modulation of GM-CSF-dependent pathways.

The clinical development of GM-CSF-targeted therapies indicates that their efficacy and safety might vary according to patient-specific factors such as disease duration, serological status, baseline inflammatory activity, and pulmonary comorbidities. Elucidating these mechanistic differences is essential to refine patient selection and reduce side effects. Overall, the distinction between ligand neutralization and receptor blockade represents a therapeutic paradigm shift in RA, providing therapeutic options beyond TNF-α, IL-6, and B cell-directed biologics for patients unresponsive to current treatments.

### 4.2. Neutralization of GM-CSF Pathway in RA: Preclinical Efficacy of Monoclonal Antibodies

Preclinical studies investigating blockade of GM-CSF signaling in RA models remain limited but provide reproducible evidence of therapeutic potential (Table 1). The mAb CAM-3003, directed against GM-CSFR, demonstrated robust anti-inflammatory activity in the mouse collagen-induced arthritis (CIA) model, where treatment significantly reduced disease severity and progression, blocked synovial inflammation, and protected against cartilage and bone damage, concomitant with decreased TNF-α and IL-1β expression in joint tissue [131]. Other studies confirmed that GM-CSF pathway inhibition attenuated CIA severity, reduced circulating Ly-6C^high^ inflammatory monocytes, and reduced synovial immune cell infiltration [132], with a dose-dependent reduction in arthritis severity associated with decreased F4/80^+^ synovial macrophages and diminished joint pathology [133]. Preclinical evidence in non-rodent species is limited; however, a protein-engineered anti-GM-CSFRα antibody (574D04) in cynomolgus monkeys demonstrated in vivo pharmacologic activity, as pretreatment with 574D04 dose-dependently suppressed GM-CSF-induced leukocyte margination and leukocytosis at doses of 1–10 mg/kg [134], supporting blockade of GM-CSF signaling. These findings highlight GM-CSF/GM-CSFR inhibition as a promising immunomodulatory strategy in RA, although preclinical data, particularly in non-human primates, remain limited.

### 4.3. Monoclonal Antibodies Against GM-CSF/GM-CSFR in RA: Clinical Trial Insights

GM-CSF and GM-CSFR have emerged as key therapeutic targets in RA, given their central role in synovial inflammation and joint damage. Numerous mAbs targeting this pathway have progressed through early- to late-phase clinical trials, demonstrating varying degrees of efficacy and safety.

Otilimab showed mixed results across trials. In the phase III ContRAst 3 study of 549 RA patients, 90 mg and 150 mg doses did not achieve significant ACR20 responses at week 12 compared with placebo, whereas sarilumab 200 mg demonstrated superior efficacy [28] (NCT04134728). In the contRAst 1 (1537 patients) and contRAst 2 (1625 patients) clinical trials, otilimab met primary endpoints with ACR20 responses, although secondary outcomes were lower than tofacitinib [135] (NCT03980483,NCT03970837). Long-term extension (contRAst X, ~3000 patients) showed a well-tolerated safety profile, with mostly moderate side events and no new safety signals [126] (NCT04333147). Phase IIa studies with weekly subcutaneous 180 mg demonstrated trends toward decreased synovitis and osteitis, though differences were not statistically significant [136] (NCT02799472).

Namilumab demonstrated more consistent efficacy. Phase II trials showed significant reductions in DAS28-CRP and improvements in ACR20/50/70 responses, with early effects visible by week 2 and MRI-confirmed reductions in synovitis, bone erosion, and bone marrow edema [137] (NCT02379091,NCT02393378). Gimsilumab and MOR103 were generally well tolerated in Phase I studies, with supportive safety for further development [138,139] (NCT01357759,NCT01023256). Plonmarlimab has finished a Phase I study, with results yet to be reported [NCT03794180], while early-phase data for TJ003234 suggest acceptable safety and GM-CSF inhibition [NCT04457856].

Mavrilimumab consistently improved DAS28-CRP and ACR responses in numerous Phase II trials. The EARTH Phase IIa study (233 patients) showed dose-dependent efficacy and early onset of improvement [140] (NCT01050998). In the EARTH EXPLORER 1 Phase IIb study, biweekly administration of 150 mg resulted in ACR20/50/70 responses, along with marked reductions in DAS28-CRP [141,142] (NCT01706926). Long-term open-label extension confirmed durable efficacy and a manageable safety profile, with no pulmonary toxicity [143,144] (NCT01712399). Pharmacokinetic analyses show dose-proportional kinetics and a half-life of approximately 13 days [143].

Finally, Table 2 provides a comparative overview of all anti-GM-CSF mAbs currently evaluated for the treatment of RA, summarizing their clinical efficacy, safety profiles, and key trial outcomes. This comparison allows for a direct assessment of therapeutic potential, dosing strategies, and long-term tolerability across the different agents.

## 5. Next-Generation Strategies for GM-CSF-Targeted Therapies in RA

### 5.1. Long-Term Safety and Immunovigilance

Although current clinical evidence supports the safety of GM-CSF mAbs in the medium term, long-term immunological consequences remain insufficiently characterized. GM-CSF is crucial for the maturation and functional competence of alveolar macrophages and plays an essential role in innate immune defense mechanisms, particularly in modulating several intracellular pathogens and fungal infections [146,147]. Prolonged pharmacovigilance through open-label extension studies and post-marketing registries will be required to detect delayed side events, including pulmonary alveolar proteinosis (PAP), uncommon opportunistic infections, and immunohematologic perturbations.

Evidence from cohorts of RA, systemic sclerosis, and COVID-19 patients treated with anti-GM-CSF agents indicates a non-trivial incidence of lower respiratory tract infections, including bacterial pneumonia [148] and invasive pulmonary aspergillosis [149]. This risk appears to be particularly elevated in individuals with pre-existing structural lung disease or concomitant corticosteroid therapy [148,149]. Importantly, subclinical surfactant accumulation and early radiographic markers of alveolar macrophage dysfunction have been documented, suggesting that pulmonary toxicity might develop insidiously before overt clinical manifestations [150,151].

The potential development of secondary PAP is of particular relevance, as GM-CSF is indispensable for surfactant catabolism and alveolar homeostasis [152]. While no definitive PAP cases have been confirmed in long-term trials to date, isolated reports of PAP-like radiological patterns underscore the need for proactive pulmonary monitoring. Moreover, hematological alterations such as monocytopenia, impaired dendritic cell differentiation, and reduced circulating myeloid precursors have been documented in pharmacodynamic studies, raising the possibility of broader myelopoietic suppression with chronic dosing [153,154].

Another important avenue of investigation involves elucidating the mechanisms underlying vaccine-induced immune responses under conditions of GM-CSF blockade. GM-CSF is a critical immunoregulatory cytokine that coordinates the interplay between innate and adaptive immune pathways by driving the differentiation, maturation, and activation of antigen-presenting cells, enhancing antigen processing and presentation, and facilitating T-cell priming and clonal expansion [155]. Consequently, pharmacologic blockade of GM-CSF signaling could theoretically attenuate vaccine responsiveness by impairing antigen presentation dynamics or downstream T-cell-mediated effector functions.

This potential immunomodulatory effect warrants comprehensive evaluation, particularly with respect to vaccines that rely on robust antigen presentation and T-cell activation, such as mRNA-based and polysaccharide formulations [156,157]. Future research should incorporate standardized and quantitative immunogenicity assays (such as measurement of neutralizing antibody titers, assessment of memory B-cell responses, and functional T-cell assays) to accurately determine the degree of post-vaccination seroprotection in patients undergoing prolonged GM-CSF inhibition. Longitudinal studies will be essential to delineate the temporal dynamics of immune recovery following treatment cessation and to establish evidence-based vaccination guidelines for this patient population [158].

### 5.2. Precision Medicine and Predictive Biomarkers

The pathogenesis of RA suggests that therapeutic response to GM-CSF inhibition will vary across patient subgroups. The development of predictive biomarkers to guide patient selection represents a critical objective. Emerging studies indicate that patients exhibiting a myeloid-dominant synovial phenotype (with enrichment of GM-CSF-responsive macrophages and monocytes) may derive greater clinical benefit [159,160]. Likewise, elevated serum concentrations of CCL17, a downstream effector chemokine triggered by GM-CSF signaling, along with increased expression of CSF2RA and CSF2RB, may serve as mechanistic biomarkers for predicting therapeutic response [161]. In parallel, accumulating preclinical data and early-phase clinical observations provide a mechanistic rationale for the co-administration of GM-CSF inhibitors with other immunomodulatory agents, such as JAK inhibitors (tofacitinib) [NCT03970837], with the aim of enhancing the depth and durability of clinical response. These combinatorial strategies are under investigation as a means to overcome cytokine network redundancy and compensatory signaling pathways that may attenuate the efficacy of GM-CSF blockade when used as monotherapy.

Comprehensive multi-omics profiling of synovial tissue, in conjunction with machine-learning-guided clustering, may allow the classification of patients into distinct GM-CSF-high and GM-CSF-low inflammatory endotypes. Prospective clinical trials incorporating adaptive enrichment designs will be necessary to confirm the validity of this biomarker-guided strategy and to optimize the risk-benefit profile at the individual level. Numerous advances in precision medicine (e.g., spatial transcriptomics, single-cell profiling, and circulating immune signatures) are expected to further refine stratification frameworks and support the rational pairing of GM-CSF inhibitors with targeted co-therapies tailored to mechanistic disease endotypes [162,163]. Well-controlled trials will also be essential to determine the optimal sequencing, dosing, and safety profile of these combination regimens.

### 5.3. Combination and Sequencing Strategies

Single-cytokine inhibition has limitations in some RA patients due to cytokine redundancy and compensatory inflammatory pathways [164]. The combination of GM-CSF inhibitors with other targeted agents (e.g., IL-6 receptor antagonists, TNF-α inhibitors, and JAK inhibitors) might provide synergistic efficacy, especially in patients with treatment-refractory disease. Combination therapy may also allow for dose reduction in individual agents, thereby potentially mitigating long-term toxicity [165].

Rational sequencing strategies must also be evaluated [166]. For instance, numerous patients with inadequate response to TNF-α inhibitors but evidence of persistent myeloid activation might transition effectively to GM-CSF blockade rather than to another TNF-α inhibitor. Clinical trials that integrate immunophenotyping and pharmacodynamic endpoints will be vital to resolving optimal treatment algorithms for real-world practice.

### 5.4. Extra-Articular Implications and Comorbidity Management

RA is a multisystem disease, and targeting GM-CSF may yield benefits beyond joint-specific inflammation. GM-CSF is implicated in the pathogenesis of RA-associated interstitial lung disease (RA-ILD) [146], one of the most severe and fatal manifestations of RA. Given that GM-CSF regulates lung-resident macrophage homeostasis and fibroblast activation, inhibition of this pathway may have therapeutic potential in patients with RA-ILD [167].

Future trials should include dedicated pulmonary endpoints, such as forced vital capacity trajectory, diffusion capacity metrics, and high-resolution computed tomography scoring. Moreover, GM-CSF blockade might alleviate systemic manifestations of RA such as fatigue, anemia of chronic disease, and symptoms driven by several myeloid cytokine networks. Incorporating validated quality-of-life measures could clarify broader patient-reported benefits.

### 5.5. Biomarker-Driven Patient Selection

The incorporation of biomarker-guided patient selection into GM-CSF-targeted therapeutic strategies holds the potential to transform RA management from empirically derived, population-based interventions toward a precision medicine paradigm [10]. Putative biomarkers (such as circulating GM-CSF concentrations, synovial expression of GM-CSF and its receptor, transcriptional signatures indicative of myeloid cell activation, specific monocyte/macrophage phenotypes, and cytokine networks) might help identify patients whose disease pathogenesis is predominantly driven by GM-CSF-mediated pathways, thus predicting therapeutic responsiveness [11]. Other approaches, such as imaging biomarkers (e.g., PET tracers indicative of macrophage activation) and pharmacogenomic variants affecting drug metabolism or immunogenicity, serve as complementary tools for patient stratification [168,169,170].

The integration of these biomarkers as enrichment or stratification variables in early-phase and adaptive clinical trials might improve signal detection, reduce unnecessary exposure in non-responders, and support the development of many companion diagnostics [171]. However, rigorous prospective validation across diverse RA populations, together with the standardization of analytical methodologies, is essential to ensure reproducibility, clinical utility, and equitable implementation in routine clinical practice [172].

### 5.6. Pediatric and Global Health Applications

The role of GM-CSF in juvenile idiopathic arthritis (JIA), especially systemic JIA and polyarticular JIA subsets, remains understood. Considering that myeloid activation is a key feature of systemic juvenile idiopathic arthritis (sJIA) and macrophage activation syndrome [173,174], GM-CSF inhibition merits investigation in pediatric populations exhibiting severe disease phenotypes.

## 6. Conclusions

Targeting GM-CSF represents a promising therapeutic strategy in the management of RA, reflecting the growing emphasis on cytokine-directed immunotherapy. Accumulating preclinical and early-phase clinical evidence supports the pathogenic role of GM-CSF in promoting synovial inflammation, joint destruction, and systemic immune dysregulation in RA. Although mAbs targeting GM-CSF or its receptor have demonstrated encouraging efficacy and acceptable safety profiles in phase I-II trials, phase III studies have so far yielded inconclusive results, preventing definitive conclusions regarding their long-term clinical utility. Accordingly, further research is needed to improve the evidence base, refine patient stratification, clarify long-term safety, and assess the potential of combination strategies to maximize therapeutic benefit.

This review advances the existing literature by providing an analysis that connects GM-CSF biology with emerging biomarker-driven and precision-medicine frameworks. Specifically, this review highlights how molecular, cellular, and clinical biomarkers may help identify patient subgroups most likely to benefit from GM-CSF inhibition and discusses the potential incorporation of GM-CSF-targeted agents into treatment algorithms. By linking therapeutic mechanisms with individualized disease profiling, this review not only summarizes current evidence but also outlines a forward-looking roadmap for the clinical translation of GM-CSF blockade in RA.

Importantly, several key questions remain unresolved and require systematic investigation. First, although short- to mid-term safety data indicate an adequate safety profile, long-term pharmacovigilance is essential to fully assess risks related to sustained GM-CSF suppression, including infection, malignancy, and alterations in tissue homeostasis. Second, reproducible criteria for patient stratification (mainly based on biomarker signatures, disease stage, or comorbid immune pathways) are still lacking and will be critical to prevent overtreatment and ensure optimal therapeutic allocation. Third, the role of GM-CSF inhibitors within combination regimens, either alongside conventional DMARDs or other targeted biologics, remains unexplored and might uncover synergistic or complementary effects.

Overall, therapies targeting GM-CSF represent a significant step forward in the development of personalized treatment strategies for RA, yet their successful translation into routine clinical practice will require the systematic resolution of these outstanding issues through rigorously designed long-term studies incorporating biomarker-based stratification and combination therapy frameworks.

## Figures and Tables

**Table 1:** Preclinical evaluation of several mAbs targeting GM-CSFR in preclinical arthritis models. Abbreviations: GM-CSFR (granulocyte-macrophage colony-stimulating factor receptor), mAb (monoclonal antibody), CIA (collagen-induced arthritis), GM-CSF (granulocyte-macrophage colony-stimulating factor), TNF-α (tumor necrosis factor-alpha), IL-1β (interleukin-1 beta), and Ly-6C^high^ (lymphocyte antigen 6C high-expressing monocytes).

Antibody Name	Target	Preclinical Model	Key Findings	References
CAM-3003	GM-CSFR	Mouse CIA model	Anti-GM-CSF mAb treatment reduced arthritis severity and progression, decreased synovial inflammation and cartilage damage, and lowered TNF-α and IL-1β levels in joint tissue	[131]
			GM-CSF blockade reduced CIA severity, circulating Ly-6C^high^ monocytes, and synovial immune cell infiltration	[132]
			Arthritis severity was reduced in a dose-dependent manner, with concomitant decreases in F4/80^+^ synovial macrophages, joint inflammation, and cartilage and bone damage	[133]
Protein-engineered anti-GM-CSFRα (574D04)	GM-CSFR	Cynomolgus monkey	Pretreatment with 574D04 dose-dependently inhibited GM-CSF-induced hematologic responses. GM-CSF triggered acute leukocyte margination and subsequent leukocytosis in controls, both of which were significantly suppressed by 574D04 at 1–10 mg/kg	[134]

**Table 2:** Therapeutic targeting of GM-CSF and GM-CSFR in RA: clinical efficacy, safety, and mechanistic insights from phase I–III trials. Abbreviations: GM-CSF (granulocyte-macrophage colony-stimulating factor), RA (rheumatoid arthritis), bDMARDs (biologic disease-modifying antirheumatic drugs), CDAI (clinical disease activity index), HAQ-DI (health assessment questionnaire disability index), ACR20 (American College of Rheumatology 20% improvement criteria), RAMRIS (rheumatoid arthritis magnetic resonance imaging score), RAMRIQ (rheumatoid arthritis magnetic resonance imaging quantitative score), OMERACT (outcome measures in rheumatology), DAS28-CRP (disease activity score in 28 joints with C-reactive protein), ACR50 (American College of Rheumatology 50% improvement criteria), ACR70 (American College of Rheumatology 70% improvement criteria), MRI (magnetic resonance imaging), DAS28 (disease activity score in 28 joints), GM-CSFR (granulocyte-macrophage colony-stimulating factor receptor), DMARD (disease-modifying antirheumatic drug), and ACR/EULAR (American College of Rheumatology/European League Against Rheumatism).

Antibody Name	Target	Key Findings	Bibliographic Reference	Clinical Trial No.
Otilimab (GSK3196165)	GM-CSF	In the phase III ContRAst 3 trial of refractory RA patients (549 patients), otilimab at 90 mg and 150 mg doses did not achieve a statistically significant ACR20 response compared with placebo at week 12, with response rates of 45% (*p* = 0.29; OR 1.38; 95% CI 0.76–2.48) and 51% (*p* = 0.06; OR 1.75; 95% CI 0.98–3.15), respectively, vs. 38% for placebo. Sarilumab 200 mg showed superior efficacy with a 57.5% ACR20 response (*p* = 0.005; OR 2.34; 95% CI 1.29–4.23). No significant improvements were observed in secondary endpoints for otilimab, and safety profiles were comparable across groups	[28]	[NCT04134728]
		In the contRAst X Phase III long-term extension trial of approximately 3000 RA patients treated with otilimab, the safety profile was sustained for up to 4 years with predominantly mild to moderate adverse events and no cases of pulmonary alveolar proteinosis, tuberculosis reactivation, or serious hypersensitivity. Adverse event rates were similar between the 90 mg and 150 mg doses, with AE incidences of 62% and 64%, respectively, and serious adverse events at 8% for both doses. The CDAI low disease activity response was maintained over time. No new safety signals were observed during long-term treatment	[126]	[NCT04333147]
		In the Phase III contRAst 1 (n = 1537) and contRAst 2 (n = 1625) trials in RA patients with inadequate response to methotrexate or bDMARDs, otilimab met the primary endpoint with significantly greater ACR20 response at 12 weeks, with contRAst 1 showing 54.7% response at 90 mg (*p* = 0.0023) and 50.9% at 150 mg (*p* = 0.0362) vs. 41.7% in placebo group. In the contRAst 2 trial, response rates were 54.9% and 54.5% for the 90 mg and 150 mg doses, respectively, compared with 32.5% for placebo (both *p* < 0.0001). Secondary endpoints (CDAI and HAQ-DI) improved but were consistently inferior to tofacitinib and the safety profile was generally well tolerated with no new safety signals reported	[135]	[NCT03980483] [NCT03970837]
		A Phase IIa clinical trial (39 patients) evaluated the effects of weekly s.c. administration of otilimab 180 mg. At week 12, otilimab produced greater reductions in RAMRIS (−1.3 ± 0.6 vs. 0.8 ± 1.2) and RAMRIQ (−1417.0 μL ± 671.5 vs. −912.3 μL ± 1405.8) synovitis scores compared with placebo, but these differences were not statistically significant for synovitis, osteitis, or bone erosion. Adverse events occurred in 39% of otilimab-treated and 36% of placebo-treated patients, most frequently cough with otilimab and extremity pain or RA symptoms with placebo. No serious adverse events or deaths were reported	[136]	[NCT02799472]
Namilumab (MT203) (AMG203)	GM-CSF	A Phase II trial (108 patients) showed that s.c. namilumab 150 mg significantly reduced disease activity in patients with moderate-to-severe RA refractory to prior therapies. At week 12, namilumab 150 mg showed a statistically significant improvement in DAS28-CRP vs. placebo (*p* = 0.005), with separation evident from week 2 (*p* < 0.05) and higher ACR50 and overall response rates. Namilumab was well tolerated with no serious safety concerns, supporting further clinical development	[137]	[NCT02379091]
		A Phase II randomized, double-blind clinical trial in 36 patients with moderate-to-severe RA receiving methotrexate demonstrated that s.c. administration of namilumab (150 mg every 2–4 weeks) significantly reduced synovitis, bone erosion, and bone marrow edema at week 24 according to OMERACT criteria. Clinical outcomes indicated improved disease activity based on DAS28-CRP (day 43, *p* = 0.0117; day 99, *p* = 0.0154) and ACR20/50/70 responses, while dynamic contrast-enhanced MRI revealed decreased synovial vascular perfusion. The treatment was well tolerated, with no safety concerns reported		[NCT02393378]
Gimsilumab (MORAb-022)	GM-CSF	Phase I (20 patients) results showed gimsilumab was well tolerated in healthy subjects and RA patients, with linear pharmacokinetics and a half-life of 9–13 days, supporting further development	[138]	[NCT01357759]
Plonmarlimab (TJ003234)	GM-CSF	Plonmarlimab has completed a Phase I study (32 patients), with results not yet published		[NCT03794180]
MOR103	GM-CSF	MOR103 was assessed in a Phase I trial (96 patients), providing initial safety and efficacy data to support larger clinical studies in active RA patients	[139]	[NCT01023256]
Mavrilimumab (CAM-3003)	GM-CSFR	In a Phase I study (32 patients), mavrilimumab showed a favorable safety and tolerability profile with single escalating intravenous doses (0.01 to 10 mg/kg) in 32 RA patients	[129]	[NCT00771420]
		EARTH phase IIa trial (233 RA patients) showed dose-dependent improvement in DAS28-CRP at 12 weeks vs. placebo. A ≥1.2 reduction in DAS28-CRP was achieved by 55.7% of patients receiving mavrilimumab vs. 34.7% in the placebo group (*p* = 0.003). The 100 mg subcutaneous biweekly cohort exhibited superior ACR response rates relative to placebo (ACR20: 69.2% vs. 40.0%, *p* = 0.005; ACR50: 30.8% vs. 12.0%, *p* = 0.021; ACR70: 17.9% vs. 4.0%, *p* = 0.03). DAS28-CRP remission (<2.6) was also significantly higher in the 100 mg group (23.1% vs. 6.7%, *p* = 0.016). Clinical improvement was evident as early as week 2 (*p* = 0.003). Mavrilimumab exhibited a favorable safety profile, with no serious pulmonary adverse events reported	[140]	[NCT01050998]
		The EARTH EXPLORER 1 phase IIb trial (233 patients) demonstrated statistically significant efficacy of mavrilimumab 150 mg s.c. biweekly. Compared to placebo, the 150 mg group had ACR20, ACR50, and ACR70 response rates of 73.4% (*p* < 0.001), 40.5% (*p* < 0.001), and 13.9% (*p* = 0.026). The drug significantly reduced DAS28-CRP scores from baseline (−1.90 vs. −0.68 for placebo; *p* < 0.001). Safety was consistent with prior studies with no unexpected serious adverse events or pulmonary toxicity reported	[141,142]	[NCT01706926]
		EARTH EXPLORER 2 study (138 patients) compared mavrilimumab 100 mg s.c. biweekly plus methotrexate vs. golimumab 50 mg s.c. every 4 weeks in 138 RA patients refractory to at least one biologic or synthetic DMARDs. Safety acceptable	[145]	[NCT01715896]
		Pharmacokinetic and exposure-efficacy modeling analyses from a Phase II study (409 patients) indicated mavrilimumab exhibits dose-proportional kinetics and an effective half-life of approximately 13 days. Efficacy was dose-dependent, with statistically significant improvements in disease activity score DAS28-CRP from baseline to week 12 across doses (150 mg: −1.90 ± 0.14; 100 mg: −1.64 ± 0.13, 30 mg: −1.37 ± 0.14; placebo: −0.68 ± 0.14; *p* < 0.001). Moreover, the proportion of patients achieving ACR20 response at week 24 was significantly higher in mavrilimumab groups (150 mg: 73.4%; 100 mg: 61.2%; 30 mg: 50.6%) vs. placebo (24.7%; *p* < 0.001).	[143]	
		Long-term open-label extension (up to 3.3 years) involving 442 RA patients on mavrilimumab plus methotrexate reported sustained reductions in DAS28-CRP scores, with 65.0% of patients achieving DAS28-CRP < 3.2 and 40.6% < 2.6 at week 122. Safety was consistent with short-term studies; adverse events were predominantly mild/moderate with no reported cases of pulmonary alveolar proteinosis or serious pulmonary toxicity	[144]	[NCT01712399]
TJ003234	GM-CSF	In a phase I/II, multicenter, randomized, double-blind, placebo-controlled trial (63 patients), single and multiple ascending doses were evaluated in adults with established RA per ACR/EULAR criteria, assessing safety, tolerability, and pharmacokinetics. Preliminary results indicate acceptable safety, predominantly mild side events, and effective GM-CSF pathway inhibition. Confirmatory efficacy and biomarker analyses are ongoing		[NCT04457856]

## Data Availability

No new data were created or analyzed in this study.

## Abbreviations

The following abbreviations are used in this manuscript:

ACPA	Anti-citrullinated protein antibody
ACR	American College of Rheumatology
ACR20	American College of Rheumatology 20% improvement criteria
ACR50	American College of Rheumatology 50% improvement criteria
ACR70	American College of Rheumatology 70% improvement criteria
Akt	Protein kinase B
anti-CCP	Anti-cyclic citrullinated peptide antibody
AP-1	Activator protein 1
APC	Antigen-presenting cell
bDMARD	Biologic disease-modifying antirheumatic drug
BCL2	B-cell lymphoma 2
BCL-XL	B-cell lymphoma-extra large
C3a	Complement component 3a
C5a	Complement component 5a
CCL17	Chemokine (C-C motif) ligand 17
CCL2	Chemokine (C-C motif) ligand 2
CCL22	Chemokine (C-C motif) ligand 22
CCL3	Chemokine (C-C motif) ligand 3
CCL5	C-C motif chemokine ligand 5
CCR2	C-C chemokine receptor type 2
CD4	Cluster of differentiation 4
CD40	Cluster of differentiation 40
CD80	Cluster of differentiation 80
CD86	Cluster of differentiation 86
CDAI	Clinical disease activity index
c-Fos	Cellular FOS proto-oncogene
CHD	Cytokine receptor homology domain
CIA	Collagen-induced arthritis
CRP	C-reactive protein
CTLA4	Cytotoxic T-lymphocyte antigen 4
CXCL10	C-X-C motif chemokine ligand 10
CXCL8	Chemokine (C-X-C motif) ligand 8
CX3CR1	CX3C chemokine receptor 1
DAS28	Disease activity score in 28 joints
DAS28-CRP	Disease activity score in 28 joints with C-reactive protein
DCE-MRI	Dynamic contrast-enhanced MRI
DMARD	Disease-modifying antirheumatic drug
Elk-1	ETS-like protein 1
ERK1/2	Extracellular signal-regulated kinase 1/2
ESR	Erythrocyte sedimentation rate
EULAR	European League Against Rheumatism
FLS	Fibroblast-like synoviocyte
GLS	Glutaminase
GM-CSF	Granulocyte-macrophage colony-stimulating factor
GM-CSFR	Granulocyte-macrophage colony-stimulating factor receptor
GM-CSFRα	Granulocyte-macrophage colony-stimulating factor receptor alpha subunit
GM-CSFRβc	Granulocyte-macrophage colony-stimulating factor receptor β common subunit
GM-DM	GM-CSF-differentiated macrophage
HAQ-DI	Health assessment questionnaire disability index
HK	Hexokinase 2
HLA-DRB1	Human leukocyte antigen-DR beta 1
IFN-γ	Interferon gamma
IgG1	Immunoglobulin G1
IKK	IκB Kinase
IL-12	Interleukin 12
IL-17	Interleukin 17
IL-17A	Interleukin 17A
IL-17F	Interleukin 17F
IL-1β	Interleukin 1 beta
IL-22	Interleukin 22
IL-23	Interleukin 23
IL-3	Interleukin 3
IL-5	Interleukin 5
IL-6	Interleukin 6
IL-8	Interleukin 8
iNOS	Inducible nitric oxide synthase
IRF4	Interferon regulatory factor 4
IRF5	Interferon regulatory factor 5
IκBα	Inhibitor of kappa B alpha
i.v.	Intravenous injection
JAK2	Janus kinase
JAK2	Janus kinase 2
JIA	Juvenile idiopathic arthritis
Ly-6C^high^	Lymphocyte antigen 6C high-expressing monocytes
M1	Classically activated macrophage phenotype
mAb	Monoclonal antibody
MAPK	Mitogen-activated protein kinase
MAPK/ERK	Mitogen-activated protein kinase/extracellular signal-regulated kinase
MCL1	Myeloid cell leukemia 1
M-CSF	Macrophage colony-stimulating factor
MEK1/2	Mitogen-activated protein kinase kinase 1/2
MHC	Major histocompatibility complex
MHC-II	Major histocompatibility complex type II
MMP	Matrix metalloproteinase
MMP-1	Matrix metalloproteinase 1
MMP-13	Matrix metalloproteinase 13
MMP-3	Matrix metalloproteinase 3
MMP-9	Matrix metalloproteinase 9
MRI	Magnetic resonance imaging
mRNA	Messenger ribonucleic acid
mTOR	Mechanistic target of rapamycin
mTORC2	Mechanistic target of rapamycin complex 2
NET	Neutrophil extracellular trap
NFATc1	Nuclear factor of activated T-cells, cytoplasmic 1
NF-κB	Nuclear factor kappa-light-chain-enhancer of activated B cells
NO	Nitric oxide
OMERACT	Outcome measures in rheumatology clinical trials
PDK1	Phosphoinositide-dependent kinase 1
PET	Positron emission tomography
PFK1	Phosphofructokinase 1
PI3K	Phosphoinositide 3-kinase
PI3K/Akt	Phosphoinositide 3-kinase/protein kinase B
PIP2	Phosphatidylinositol 4,5-bisphosphate
PIP3	Phosphatidylinositol 3,4,5-trisphosphate
PK	Pharmacokinetics
PTPN22	Protein tyrosine phosphatase non-receptor type 22
RA	Rheumatoid arthritis
RA-ILD	Rheumatoid arthritis-associated interstitial lung disease
Raf-1	Rapidly accelerated fibrosarcoma 1
RAMRIS	Rheumatoid arthritis magnetic resonance imaging score
RAMRIQ	Rheumatoid arthritis magnetic resonance imaging quantitative
RANK	Receptor activator of nuclear factor κB
RANKL	Receptor activator of nuclear factor κB ligand
Ras	Rat sarcoma
RF	Rheumatoid factor
ROS	Reactive oxygen species
s.c.	Subcutaneous injection
SH2	Src homology 2
sJIA	Systemic juvenile idiopathic arthritis
SOCS3	Suppressor of cytokine signaling 3
STAT	Signal transducer and activator of transcription
STAT4	Signal transducer and activator of transcription 4
STAT5	Signal transducer and activator of transcription 5
Th	T helper lymphocyte
Th1	T helper 1 cell
Th17	T helper 17 cell
TNF-α	Tumor necrosis factor alpha
Treg	Regulatory T cell

## References

[1] Gao Y., Zhang Y., Liu X. (2024). Rheumatoid arthritis: Pathogenesis and therapeutic advances. MedComm.

[2] Scherer H.U., Häupl T., Burmester G.R. (2020). The etiology of rheumatoid arthritis. J. Autoimmun..

[3] Romão V.C., Fonseca J.E. (2021). Etiology and Risk Factors for Rheumatoid Arthritis: A State-of-the-Art Review. Front. Med..

[4] Weyand C.M., Goronzy J.J. (2021). The immunology of rheumatoid arthritis. Nat. Immunol..

[5] Edilova M.I., Akram A., Abdul-Sater A.A. (2021). Innate immunity drives pathogenesis of rheumatoid arthritis. Biomed. J..

[6] Yamada H. (2022). Adaptive immunity in the joint of rheumatoid arthritis. Immunol. Med..

[7] Brzustewicz E., Bryl E. (2015). The role of cytokines in the pathogenesis of rheumatoid arthritis—Practical and potential application of cytokines as biomarkers and targets of personalized therapy. Cytokine.

[8] Mateen S., Zafar A., Moin S., Khan A.Q., Zubair S. (2016). Understanding the role of cytokines in the pathogenesis of rheumatoid arthritis. Clin. Chim. Acta.

[9] Fujimoto S., Niiro H. (2025). Pathogenic Role of Cytokines in Rheumatoid Arthritis. J. Clin. Med..

[10] Fuentelsaz-Romero S., Cuervo A., Estrada-Capetillo L., Celis R., García-Campos R., Ramírez J., Sastre S., Samaniego R., Puig-Kröger A., Cañete J.D. (2021). GM-CSF Expression and Macrophage Polarization in Joints of Undifferentiated Arthritis Patients Evolving to Rheumatoid Arthritis or Psoriatic Arthritis. Front. Immunol..

[11] Su J., Hu W., Ding Y., Zhang P., Li T., Liu S., Xing L. (2024). Serum GM-CSF level is a predictor of treatment response to tocilizumab in rheumatoid arthritis patients: A prospective observational cohort study. Arthritis Res. Ther..

[12] Shiomi A., Usui T., Mimori T. (2016). GM-CSF as a therapeutic target in autoimmune diseases. Inflamm. Regen..

[13] Lee K.M.C., Achuthan A.A., Hamilton J.A. (2020). GM-CSF: A Promising Target in Inflammation and Autoimmunity. Immunotargets Ther..

[14] Woodcock J.M., McClure B.J., Stomski F.C., Elliott M.J., Bagley C.J., Lopez A.F. (1997). The human granulocyte-macrophage colony-stimulating factor (GM-CSF) receptor exists as a preformed receptor complex that can be activated by GM-CSF, interleukin-3, or interleukin-5. Blood.

[15] Hansen G., Hercus T.R., McClure B.J., Stomski F.C., Dottore M., Powell J., Ramshaw H., Woodcock J.M., Xu Y., Guthridge M. (2008). The structure of the GM-CSF receptor complex reveals a distinct mode of cytokine receptor activation. Cell.

[16] Hu X., Li J., Fu M., Zhao X., Wang W. (2021). The JAK/STAT signaling pathway: From bench to clinic. Signal Transduct. Target. Ther..

[17] Nagenborg J., Jin H., Ruder A.V., Temmerman L., Mees B., Schalkwijk C., Müller-Klieser D., Berg T., Goossens P., Donners M.M.P.C. (2023). GM-CSF-activated STAT5A regulates macrophage functions and inflammation in atherosclerosis. Front. Immunol..

[18] Larbi A., Douziech N., Fortin C., Linteau A., Dupuis G., Fulop T. (2005). The role of the MAPK pathway alterations in GM-CSF modulated human neutrophil apoptosis with aging. Immun. Ageing.

[19] Han N.-R., Park H.-J., Moon P.-D. (2022). Resveratrol Downregulates Granulocyte-Macrophage Colony-Stimulating Factor-Induced Oncostatin M Production through Blocking of PI3K/Akt/NF-κB Signal Cascade in Neutrophil-like Differentiated HL-60 Cells. Curr. Issues Mol. Biol..

[20] Chen J., Zhan M., Zhao Y., Xu H., Feng F., Bai Z., Zhang K., Fu L., Wang F., Cheng Y. (2025). GM-CSF potentiates macrophages to retain an inflammatory feature from their circulating monocyte precursors in rheumatoid arthritis. J. Transl. Med..

[21] Lotfi N., Thome R., Rezaei N., Zhang G.X., Rezaei A., Rostami A., Esmaeil N. (2019). Roles of GM-CSF in the Pathogenesis of Autoimmune Diseases: An Update. Front. Immunol..

[22] Hamilton J.A. (2020). GM-CSF in inflammation. J. Exp. Med..

[23] Domańska-Poboża J., Wisłowska M. (2025). Evolving strategies in the treatment of rheumatoid arthritis: A historical perspective. Reumatologia.

[24] Zhang Q., McDermott G.C., Juge P.A., Chang S.H., Vanni K.M., Qian G., Bade K.J., Mueller K.T., Kowalski E.N., Saavedra A.A. (2024). Disease-modifying antirheumatic drugs and risk of incident interstitial lung disease among patients with rheumatoid arthritis: A systematic review and meta-analysis. Semin. Arthritis Rheum..

[25] Smolen J.S., Landewé R.B.M., Bergstra S.A., Kerschbaumer A., Sepriano A., Aletaha D., Caporali R., Edwards C.J., Hyrich K.L., Pope J.E. (2023). EULAR recommendations for the management of rheumatoid arthritis with synthetic and biological disease-modifying antirheumatic drugs: 2022 update. Ann. Rheum. Dis..

[26] Crotti C., Agape E., Becciolini A., Biggioggero M., Favalli E.G. (2019). Targeting Granulocyte-Monocyte Colony-Stimulating Factor Signaling in Rheumatoid Arthritis: Future Prospects. Drugs.

[27] Bykerk V.P. (2020). The efficacy and safety of targeting GM-CSF in arthritis. Lancet Rheumatol..

[28] Taylor P.C., Weinblatt M.E., McInnes I.B., Atsumi T., Strand V., Takeuchi T., Bracher M., Brooks D., Davies J., Goode C. (2023). Anti-GM-CSF otilimab versus sarilumab or placebo in patients with rheumatoid arthritis and inadequate response to targeted therapies: A phase III randomised trial (contRAst 3). Ann. Rheum. Dis..

[29] Weinblatt M.E., McInnes I.B., Kremer J.M., Miranda P., Vencovsky J., Guo X., White W.I., Ryan P.C., Godwood A., Albulescu M. (2018). A Randomized Phase IIb Study of Mavrilimumab and Golimumab in Rheumatoid Arthritis. Arthritis Rheumatol..

[30] Buckley C.D., Simón-Campos J.A., Zhdan V., Becker B., Davy K., Fisheleva E., Gupta A., Hawkes C., Inman D., Layton M. (2020). Efficacy, patient-reported outcomes, and safety of the anti-granulocyte macrophage colony-stimulating factor antibody otilimab (GSK3196165) in patients with rheumatoid arthritis: A randomised, phase 2b, dose-ranging study. Lancet Rheumatol..

[31] Genovese M.C., Buckley C.D., Saurigny D., Schett G., Davy K., Gupta A., Smith J.E., Patel J., Tak P.P. (2021). Targeting GM-CSF in rheumatological conditions: Risk of PAP—Authors’ reply. Lancet Rheumatol..

[32] Zhang Z., Gao X., Liu S., Wang Q., Wang Y., Hou S., Wang J., Zhang Y. (2025). Global, regional, and national epidemiology of rheumatoid arthritis among people aged 20–54 years from 1990 to 2021. Sci. Rep..

[33] Maranini B., Bortoluzzi A., Silvagni E., Govoni M. (2022). Focus on Sex and Gender: What We Need to Know in the Management of Rheumatoid Arthritis. J. Pers. Med..

[34] Scott D.L., Steer S. (2007). The course of established rheumatoid arthritis. Best Pract. Res. Clin. Rheumatol..

[35] Rawla P. (2019). Cardiac and vascular complications in rheumatoid arthritis. Reumatologia.

[36] Wu D., Luo Y., Li T., Zhao X., Lv T., Fang G., Ou P., Li H., Luo X., Huang A. (2022). Systemic complications of rheumatoid arthritis: Focus on pathogenesis and treatment. Front. Immunol..

[37] Dedmon L.E. (2020). The genetics of rheumatoid arthritis. Rheumatology.

[38] Wysocki T., Olesińska M., Paradowska-Gorycka A. (2020). Current Understanding of an Emerging Role of HLA-DRB1 Gene in Rheumatoid Arthritis–From Research to Clinical Practice. Cells.

[39] Mustelin T., Bottini N., Stanford S.M. (2019). The Contribution of PTPN22 to Rheumatic Disease. Arthritis Rheumatol..

[40] Bravo-Villagra K.M., Muñoz-Valle J.F., Baños-Hernández C.J., Cerpa-Cruz S., Navarro-Zarza J.E., Parra-Rojas I., Aguilar-Velázquez J.A., García-Arellano S., López-Quintero A. (2024). *STAT4* Gene Variant *rs7574865* Is Associated with Rheumatoid Arthritis Activity and Anti-CCP Levels in the Western but Not in the Southern Population of Mexico. Genes.

[41] Zhou C., Gao S., Yuan X., Shu Z., Li S., Sun X., Xiao J., Liu H. (2021). Association between *CTLA-4* gene polymorphism and risk of rheumatoid arthritis: A meta-analysis. Aging.

[42] Alfredsson L., Olsson T. (2019). Lifestyle and Environmental Factors in Multiple Sclerosis. Cold Spring Harb. Perspect. Med..

[43] Li Y., Guo R., Oduro P.K., Sun T., Chen H., Yi Y., Zeng W., Wang Q., Leng L., Yang L. (2022). The Relationship Between Porphyromonas Gingivalis and Rheumatoid Arthritis: A Meta-Analysis. Front. Cell Infect. Microbiol..

[44] Konig M.F., Abusleme L., Reinholdt J., Palmer R.J., Teles R.P., Sampson K., Rosen A., Nigrovic P.A., Sokolove J., Giles J.T. (2016). Aggregatibacter actinomycetemcomitans-induced hypercitrullination links periodontal infection to autoimmunity in rheumatoid arthritis. Sci. Transl. Med..

[45] Jiang L.Q., Zhang R.D., Musonye H.A., Zhao H.Y., He Y.S., Zhao C.N., He T., Tian T., Gao Z.X., Fang Y. (2024). Hormonal and reproductive factors in relation to the risk of rheumatoid arthritis in women: A prospective cohort study with 223 526 participants. RMD Open.

[46] Shuai Z., Zheng S., Wang K., Wang J., Leung P.S.C., Xu B. (2022). Reestablish immune tolerance in rheumatoid arthritis. Front. Immunol..

[47] Carlé C., Degboe Y., Ruyssen-Witrand A., Arleevskaya M.I., Clavel C., Renaudineau Y. (2023). Characteristics of the (Auto)Reactive T Cells in Rheumatoid Arthritis According to the Immune Epitope Database. Int. J. Mol. Sci..

[48] van Loosdregt J., Rossetti M., Spreafico R., Moshref M., Olmer M., Williams G.W., Kumar P., Copeland D., Pischel K., Lotz M. (2016). Increased autophagy in CD4^+^ T cells of rheumatoid arthritis patients results in T-cell hyperactivation and apoptosis resistance. Eur. J. Immunol..

[49] Luo P., Wang P., Xu J., Hou W., Xu P., Xu K., Liu L. (2022). Immunomodulatory role of T helper cells in rheumatoid arthritis: A comprehensive research review. Bone Jt. Res..

[50] Yan S., Kotschenreuther K., Deng S., Kofler D.M. (2022). Regulatory T cells in rheumatoid arthritis: Functions, development, regulation, and therapeutic potential. Cell. Mol. Life Sci..

[51] Bartok B., Firestein G.S. (2010). Fibroblast-like synoviocytes: Key effector cells in rheumatoid arthritis. Immunol. Rev..

[52] Tsaltskan V., Firestein G.S. (2022). Targeting fibroblast-like synoviocytes in rheumatoid arthritis. Curr. Opin. Pharmacol..

[53] Brescia A.C., Simonds M.M., Sullivan K.E., Rose C.D. (2017). Secretion of pro-inflammatory cytokines and chemokines and loss of regulatory signals by fibroblast-like synoviocytes in juvenile idiopathic arthritis. Proteom. Clin. Appl..

[54] Sokolova M.V., Schett G., Steffen U. (2022). Autoantibodies in Rheumatoid Arthritis: Historical Background and Novel Findings. Clin. Rev. Allergy Immunol..

[55] Banda N.K., Hyatt S., Antonioli A.H., White J.T., Glogowska M., Takahashi K., Merkel T.J., Stahl G.L., Mueller-Ortiz S., Wetsel R. (2012). Role of C3a receptors, C5a receptors, and complement protein C6 deficiency in collagen antibody-induced arthritis in mice. J. Immunol..

[56] Youinou P., Mackenzie L., Katsikis P., Merdrignac G., Isenberg D.A., Tuaillon N., Lamour A., Le Goff P., Jouquan J., Drogou A. (1990). The relationship between CD5-expressing B lymphocytes and serologic abnormalities in rheumatoid arthritis patients and their relatives. Arthritis Rheum..

[57] McGrath S., Grimstad K., Thorarinsdottir K., Forslind K., Glinatsi D., Leu Agelii M., Aranburu A., Sundell T., Jonsson C.A., Camponeschi A. (2024). Correlation of Professional Antigen-Presenting Tbet^+^CD11c^+^ B Cells With Bone Destruction in Untreated Rheumatoid Arthritis. Arthritis Rheumatol..

[58] Deng Y., Li J., Wu R. (2025). Neutrophils in Rheumatoid Arthritis Synovium: Implications on Disease Activity and Inflammation State. J. Inflamm. Res..

[59] Zheng Y., Wei K., Jiang P., Zhao J., Shan Y., Shi Y., Zhao F., Chang C., Li Y., Zhou M. (2024). Macrophage polarization in rheumatoid arthritis: Signaling pathways, metabolic reprogramming, and crosstalk with synovial fibroblasts. Front. Immunol..

[60] Chen L., Lu Y., Chu Y., Xie J., Ding W., Wang F. (2013). Tissue factor expression in rheumatoid synovium: A potential role in pannus invasion of rheumatoid arthritis. Acta Histochem..

[61] Tanaka S. (2019). RANKL is a therapeutic target of bone destruction in rheumatoid arthritis. F1000Research.

[62] De Leon-Oliva D., Barrena-Blázquez S., Jiménez-Álvarez L., Fraile-Martinez O., García-Montero C., López-González L., Torres-Carranza D., García-Puente L.M., Carranza S.T., Álvarez-Mon M.Á. (2023). The RANK–RANKL–OPG System: A Multifaceted Regulator of Homeostasis, Immunity, and Cancer. Medicina.

[63] Rolph D., Das H. (2020). Transcriptional Regulation of Osteoclastogenesis: The Emerging Role of KLF2. Front. Immunol..

[64] DI Matteo A., Emery P. (2024). Rheumatoid arthritis: A review of the key clinical features and ongoing challenges of the disease. Panminerva Med..

[65] Zimba O., Baimukhamedov C., Kocyigit B.F. (2025). Late-onset rheumatoid arthritis: Clinical features, diagnostic challenges, and treatment approaches. Rheumatol. Int..

[66] Pavlov-Dolijanovic S., Bogojevic M., Nozica-Radulovic T., Radunovic G., Mujovic N. (2023). Elderly-Onset Rheumatoid Arthritis: Characteristics and Treatment Options. Medicina.

[67] Chmielewski G., Majewski M.S., Kuna J., Mikiewicz M., Krajewska-Włodarczyk M. (2023). Fatigue in Inflammatory Joint Diseases. Int. J. Mol. Sci..

[68] Debreova M., Culenova M., Smolinska V., Nicodemou A., Csobonyeiova M., Danisovic L. (2022). Rheumatoid arthritis: From synovium biology to cell-based therapy. Cytotherapy.

[69] Cojocaru M., Cojocaru I.M., Silosi I., Vrabie C.D., Tanasescu R. (2010). Extra-articular Manifestations in Rheumatoid Arthritis. Maedica.

[70] Sanghavi N., Ingrassia J.P., Korem S., Ash J., Pan S., Wasserman A. (2024). Cardiovascular Manifestations in Rheumatoid Arthritis. Cardiol. Rev..

[71] Aletaha D., Smolen J.S. (2018). Diagnosis and Management of Rheumatoid Arthritis: A Review. JAMA.

[72] Kay J., Upchurch K.S. (2012). ACR/EULAR 2010 rheumatoid arthritis classification criteria. Rheumatology.

[73] Aggarwal R., Liao K., Nair R., Ringold S., Costenbader K.H. (2009). Anti-citrullinated peptide antibody assays and their role in the diagnosis of rheumatoid arthritis. Arthritis Rheum..

[74] Kgoebane K., Ally M.M.T.M., Duim-Beytell M.C., Suleman F.E. (2018). The role of imaging in rheumatoid arthritis. SA J. Radiol..

[75] Hercus T.R., Thomas D., Guthridge M.A., Ekert P.G., King-Scott J., Parker M.W., Lopez A.F. (2009). The granulocyte-macrophage colony-stimulating factor receptor: Linking its structure to cell signaling and its role in disease. Blood.

[76] Weng S., Zhang D.-E. (2015). The GM-CSF Receptor Alpha Chain (CSF2RA) Functions As a Novel Ligand-Independent Tumor Suppressor in t(8;21) AML. Blood.

[77] Mirza S., Walker A., Chen J., Murphy J.M., Young I.G. (2010). The Ig-like domain of human GM-CSF receptor alpha plays a critical role in cytokine binding and receptor activation. Biochem. J..

[78] Pundavela J., Hall A., Dinglasan S.A., Choi K., Rizvi T.A., Trapnell B.C., Wu J., Ratner N. (2025). Granulocyte-Macrophage Colony Stimulating Factor Receptor Contributes to Plexiform Neurofibroma Initiation. Cancers.

[79] Carr P.D., Gustin S.E., Church A.P., Murphy J.M., Ford S.C., Mann D.A., Woltring D.M., Walker I., Ollis D.L., Young I.G. (2001). Structure of the complete extracellular domain of the common beta subunit of the human GM-CSF, IL-3, and IL-5 receptors reveals a novel dimer configuration. Cell.

[80] Zsiros V., Katz S., Doczi N., Kiss A.L. (2019). Endocytosis of GM-CSF receptor β is essential for signal transduction regulating mesothelial-macrophage transition. Biochim. Biophys. Acta Mol. Cell Res..

[81] Zhao Y., Wagner F., Frank S.J., Kraft A.S. (1995). The amino-terminal portion of the JAK2 protein kinase is necessary for binding and phosphorylation of the granulocyte-macrophage colony-stimulating factor receptor beta c chain. J. Biol. Chem..

[82] Liu R.Y., Fan C., Garcia R., Jove R., Zuckerman K.S. (1999). Constitutive activation of the JAK2/STAT5 signal transduction pathway correlates with growth factor independence of megakaryocytic leukemic cell lines. Blood.

[83] Kimura A., Rieger M.A., Simone J.M., Chen W., Wickre M.C., Zhu B.M., Hoppe P.S., O’Shea J.J., Schroeder T., Hennighausen L. (2009). The transcription factors STAT5A/B regulate GM-CSF-mediated granulopoiesis. Blood.

[84] Rumore-Maton B., Elf J., Belkin N., Stutevoss B., Seydel F., Garrigan E., Litherland S.A. (2008). M-CSF and GM-CSF regulation of STAT5 activation and DNA binding in myeloid cell differentiation is disrupted in nonobese diabetic mice. Clin. Dev. Immunol..

[85] Vázquez Marrero V.R., Dresler M., Haggadone M.D., Lu A., Shin S. (2025). GM-CSF engages multiple signaling pathways to enhance pro-inflammatory cytokine responses in human monocytes during *Legionella* infection. Infect. Immun..

[86] López-Navarro B., Simón-Fuentes M., Ríos I., Schiaffino M.T., Sanchez A., Torres-Torresano M., Nieto-Valle A., Castrejón I., Puig-Kröger A. (2024). Macrophage re-programming by JAK inhibitors relies on MAFB. Cell Mol. Life Sci..

[87] Boyer S., Lee H.J., Steele N., Zhang L., Sajjakulnukit P., Andren A., Ward M.H., Singh R., Basrur V., Zhang Y. (2022). Multiomic characterization of pancreatic cancer-associated macrophage polarization reveals deregulated metabolic programs driven by the GM-CSF-PI3K pathway. Elife.

[88] Jücker M., Feldman R.A. (1995). Identification of a new adapter protein that may link the common beta subunit of the receptor for granulocyte/macrophage colony-stimulating factor, interleukin (IL)-3, and IL-5 to phosphatidylinositol 3-kinase. J. Bol. Chem..

[89] Mafi S., Mansoori B., Taeb S., Sadeghi H., Abbasi R., Cho W.C., Rostamzadeh D. (2022). mTOR-Mediated Regulation of Immune Responses in Cancer and Tumor Microenvironment. Front. Immunol..

[90] de Carvalho Oliveira V., Tatsiy O., McDonald P.P. (2023). Phosphoinositol 3-kinase-driven NET formation involves different isoforms and signaling partners depending on the stimulus. Front. Immunol..

[91] Kolonics A., Apáti A., Jánossy J., Brózik A., Gáti R., Schaefer A., Magócsi M. (2001). Activation of Raf/ERK1/2 MAP kinase pathway is involved in GM-CSF-induced proliferation and survival but not in erythropoietin-induced differentiation of TF-1 cells. Cell Signal..

[92] Schallenberg M., Charalambous P., Thanos S. (2009). GM-CSF regulates the ERK1/2 pathways and protects injured retinal ganglion cells from induced death. Exp. Eye Res..

[93] Parajuli B., Sonobe Y., Kawanokuchi J., Doi Y., Noda M., Takeuchi H., Mizuno T., Suzumura A. (2012). GM-CSF increases LPS-induced production of proinflammatory mediators via upregulation of TLR4 and CD14 in murine microglia. J. Neuroinflamm..

[94] Hu N., Qiu Y., Dong F. (2015). Role of Erk1/2 signaling in the regulation of neutrophil versus monocyte development in response to G-CSF and M-CSF. J. Biol. Chem..

[95] Rodriguez R.M., Suarez-Alvarez B., Lavín J.L., Ascensión A.M., Gonzalez M., Lozano J.J., Raneros A.B., Bulnes P.D., Vidal-Castiñeira J.R., Huidobro C. (2019). Signal Integration and Transcriptional Regulation of the Inflammatory Response Mediated by the GM-/M-CSF Signaling Axis in Human Monocytes. Cell Rep..

[96] Ebner K., Bandion A., Binder B.R., de Martin R., Schmid J.A. (2003). GMCSF activates NF-kappaB via direct interaction of the GMCSF receptor with IkappaB kinase beta. Blood.

[97] Guerrero P., Bono C., Sobén M., Guiu A., Cheng Q.J., Gil M.L., Yáñez A. (2024). GM-CSF receptor expression determines opposing innate memory phenotypes at different stages of myelopoiesis. Blood.

[98] Cook A.D., Louis C., Robinson M.J., Saleh R., Sleeman M.A., Hamilton J.A. (2016). Granulocyte macrophage colony-stimulating factor receptor α expression and its targeting in antigen-induced arthritis and inflammation. Arthritis Res. Ther..

[99] Yoshitomi H. (2019). Regulation of Immune Responses and Chronic Inflammation by Fibroblast-Like Synoviocytes. Front. Immunol..

[100] Tu J., Hong W., Zhang P., Wang X., Körner H., Wei W. (2018). Ontology and Function of Fibroblast-Like and Macrophage-Like Synoviocytes: How Do They Talk to Each Other and Can They Be Targeted for Rheumatoid Arthritis Therapy?. Front. Immunol..

[101] Xiang C., Li H., Tang W. (2023). Targeting CSF-1R represents an effective strategy in modulating inflammatory diseases. Pharmacol. Res..

[102] Zhan Y., Lew A.M., Chopin M. (2019). The Pleiotropic Effects of the GM-CSF Rheostat on Myeloid Cell Differentiation and Function: More Than a Numbers Game. Front. Immunol..

[103] Pérez S., Rius-Pérez S. (2022). Macrophage Polarization and Reprogramming in Acute Inflammation: A Redox Perspective. Antioxidants.

[104] Lotfi N., Zhang G.X., Esmaeil N., Rostami A. (2020). Evaluation of the effect of GM-CSF blocking on the phenotype and function of human monocytes. Sci. Rep..

[105] Subramanian Vignesh K., Landero Figueroa J.A., Porollo A., Caruso J.A., Deepe G.S. (2013). Granulocyte macrophage-colony stimulating factor induced Zn sequestration enhances macrophage superoxide and limits intracellular pathogen survival. Immunity.

[106] Takeuchi Y., Hirota K., Sakaguchi S. (2019). Synovial Tissue Inflammation Mediated by Autoimmune T Cells. Front. Immunol..

[107] Fuhler G.M., Cadwallader K.A., Knol G.J., Chilvers E.R., Drayer A.L., Vellenga E. (2004). Disturbed granulocyte macrophage-colony stimulating factor priming of phosphatidylinositol 3,4,5-trisphosphate accumulation and Rac activation in fMLP-stimulated neutrophils from patients with myelodysplasia. J. Leukoc. Biol..

[108] Zhang Y., McCluskey K., Fujii K., Wahl L.M. (1998). Differential regulation of monocyte matrix metalloproteinase and TIMP-1 production by TNF-alpha, granulocyte-macrophage CSF, and IL-1 beta through prostaglandin-dependent and -independent mechanisms. J. Immunol..

[109] Schreck R., Baeuerle P.A. (1990). NF-kappa B as inducible transcriptional activator of the granulocyte-macrophage colony-stimulating factor gene. Mol. Cell. Biol..

[110] Weiss M., Blazek K., Byrne A.J., Perocheau D.P., Udalova I.A. (2013). IRF5 is a specific marker of inflammatory macrophages in vivo. Mediat. Inflamm..

[111] Helft J., Böttcher J.P., Chakravarty P., Zelenay S., Huotari J., Schraml B.U., Goubau D., Reis e Sousa C. (2016). Alive but Confused: Heterogeneity of CD11c(+) MHC Class II(+) Cells in GM-CSF Mouse Bone Marrow Cultures. Immunity.

[112] Mashima H., Zhang R., Kobayashi T., Hagiya Y., Tsukamoto H., Liu T., Iwama T., Yamamoto M., Lin C., Nakatsuka R. (2020). Generation of GM-CSF-producing antigen-presenting cells that induce a cytotoxic T cell-mediated antitumor response. Oncoimmunology.

[113] Korniotis S., Saichi M., Trichot C., Hoffmann C., Amblard E., Viguier A., Grondin S., Noel F., Mattoo H., Soumelis V. (2022). GM-CSF-activated human dendritic cells promote type 1 T follicular helper cell polarization in a CD40-dependent manner. J. Cell Sci..

[114] Gilmour B.C., Corthay A., Øynebråten I. (2024). High production of IL-12 by human dendritic cells stimulated with combinations of pattern-recognition receptor agonists. npj Vaccines.

[115] Hirota K., Hashimoto M., Ito Y., Matsuura M., Ito H., Tanaka M., Watanabe H., Kondoh G., Tanaka A., Yasuda K. (2018). Autoimmune Th17 Cells Induced Synovial Stromal and Innate Lymphoid Cell Secretion of the Cytokine GM-CSF to Initiate and Augment Autoimmune Arthritis. Immunity.

[116] Spiekermann K., Roesler J., Emmendoerffer A., Elsner J., Welte K. (1997). Functional features of neutrophils induced by G-CSF and GM-CSF treatment: Differential effects and clinical implications. Leukemia.

[117] Chao J.R., Wang J.M., Lee S.F., Peng H.W., Lin Y.H., Chou C.H., Li J.C., Huang H.M., Chou C.K., Kuo M.L. (1998). mcl-1 is an immediate-early gene activated by the granulocyte-macrophage colony-stimulating factor (GM-CSF) signaling pathway and is one component of the GM-CSF viability response. Mol. Cell. Biol..

[118] He K., Liu X., Hoffman R.D., Shi R.Z., Lv G.Y., Gao J.L. (2022). G-CSF/GM-CSF-induced hematopoietic dysregulation in the progression of solid tumors. FEBS Open Bio.

[119] Chen J., Cao Y., Xiao J., Hong Y., Zhu Y. (2024). The emerging role of neutrophil extracellular traps in the progression of rheumatoid arthritis. Front. Immunol..

[120] Bhattacharya P., Thiruppathi M., Elshabrawy H.A., Alharshawi K., Kumar P., Prabhakar B.S. (2015). GM-CSF: An immune modulatory cytokine that can suppress autoimmunity. Cytokine.

[121] Singh P., González-Ramos S., Mojena M., Rosales-Mendoza C.E., Emami H., Swanson J., Morss A., Fayad Z.A., Rudd J.H., Gelfand J. (2016). GM-CSF Enhances Macrophage Glycolytic Activity In Vitro and Improves Detection of Inflammation In Vivo. J. Nucl. Med..

[122] Sun H.W., Wu W.C., Chen H.T., Xu Y.T., Yang Y.Y., Chen J., Yu X.J., Wang Z., Shuang Z.Y., Zheng L. (2021). Glutamine Deprivation Promotes the Generation and Mobilization of MDSCs by Enhancing Expression of G-CSF and GM-CSF. Front. Immunol..

[123] Guo X., Wang S., Godwood A., Close D., Ryan P.C., Roskos L.K., White W.I. (2019). Pharmacodynamic biomarkers and differential effects of TNF- and GM-CSF-targeting biologics in rheumatoid arthritis. Int. J. Rheum. Dis..

[124] Senolt L. (2019). Emerging therapies in rheumatoid arthritis: Focus on monoclonal antibodies. F1000Research.

[125] Kumar A., Taghi Khani A., Sanchez Ortiz A., Swaminathan S. (2022). GM-CSF: A Double-Edged Sword in Cancer Immunotherapy. Front. Immunol..

[126] Weinblatt M.E., Taylor P.C., McInnes I.B., Atsumi T., Strand V., Takeuchi T., Bracher M., Brooks D., Curtis P., Gupta A. (2025). Long-term safety and efficacy of anti-GM-CSF otilimab in patients with rheumatoid arthritis: Long-term extension of three phase 3 randomised trials (contRAst X). BMJ Open.

[127] Huizinga T.W., Batalov A., Stoilov R., Lloyd E., Wagner T., Saurigny D., Souberbielle B., Esfandiari E. (2017). Phase 1b randomized, double-blind study of namilumab, an anti-granulocyte macrophage colony-stimulating factor monoclonal antibody, in mild-to-moderate rheumatoid arthritis. Arthritis Res. Ther..

[128] Corbera-Bellalta M., Alba-Rovira R., Muralidharan S., Espígol-Frigolé G., Ríos-Garcés R., Marco-Hernández J., Denuc A., Kamberovic F., Pérez-Galán P., Joseph A. (2022). Blocking GM-CSF receptor α with mavrilimumab reduces infiltrating cells, pro-inflammatory markers and neoangiogenesis in ex vivo cultured arteries from patients with giant cell arteritis. Ann. Rheum. Dis..

[129] Burmester G.R., Feist E., Sleeman M.A., Wang B., White B., Magrini F. (2011). Mavrilimumab, a human monoclonal antibody targeting GM-CSF receptor-α, in subjects with rheumatoid arthritis: A randomised, double-blind, placebo-controlled, phase I, first-in-human study. Ann. Rheum. Dis..

[130] Davda J.P., Hansen R.J. (2010). Properties of a general PK/PD model of antibody-ligand interactions for therapeutic antibodies that bind to soluble endogenous targets. MAbs.

[131] Cook A.D., Braine E.L., Campbell I.K., Rich M.J., Hamilton J.A. (2001). Blockade of collagen-induced arthritis post-onset by antibody to granulocyte-macrophage colony-stimulating factor (GM-CSF): Requirement for GM-CSF in the effector phase of disease. Arthritis Res..

[132] Cook A.D., Turner A.L., Braine E.L., Pobjoy J., Lenzo J.C., Hamilton J.A. (2011). Regulation of systemic and local myeloid cell subpopulations by bone marrow cell-derived granulocyte-macrophage colony-stimulating factor in experimental inflammatory arthritis. Arthritis Rheum..

[133] Greven D.E., Cohen E.S., Gerlag D.M., Campbell J., Woods J., Davis N., van Nieuwenhuijze A., Lewis A., Heasmen S., McCourt M. (2015). Preclinical characterisation of the GM-CSF receptor as a therapeutic target in rheumatoid arthritis. Ann. Rheum. Dis..

[134] Minter R.R., Cohen E.S., Wang B., Liang M., Vainshtein I., Rees G., Eghobamien L., Harrison P., Sims D.A., Matthews C. (2013). Protein engineering and preclinical development of a GM-CSF receptor antibody for the treatment of rheumatoid arthritis. Br. J. Pharmacol..

[135] Fleischmann R.M., van der Heijde D., Strand V., Atsumi T., McInnes I.B., Takeuchi T., Taylor P.C., Bracher M., Brooks D., Davies J. (2023). Anti-GM-CSF otilimab versus tofacitinib or placebo in patients with active rheumatoid arthritis and an inadequate response to conventional or biologic DMARDs: Two phase 3 randomised trials (contRAst 1 and contRAst 2). Ann. Rheum. Dis..

[136] Genovese M.C., Berkowitz M., Conaghan P.G., Peterfy C., Davy K., Fisheleva E., Gupta A., Inman D., Janiczek R., Layton M. (2020). MRI of the joint and evaluation of the granulocyte-macrophage colony-stimulating factor-CCL17 axis in patients with rheumatoid arthritis receiving otilimab: A phase 2a randomised mechanistic study. Lancet Rheumatol..

[137] Taylor P.C., Saurigny D., Vencovsky J., Takeuchi T., Nakamura T., Matsievskaia G., Hunt B., Wagner T., Souberbielle B., NEXUS Study Group (2019). Efficacy and safety of namilumab, a human monoclonal antibody against granulocyte-macrophage colony-stimulating factor (GM-CSF) ligand in patients with rheumatoid arthritis (RA) with either an inadequate response to background methotrexate therapy or an inadequate response or intolerance to an anti-TNF (tumour necrosis factor) biologic therapy: A randomized, controlled trial. Arthritis Res. Ther..

[138] Kivitz A., Hazan L., Hoffman K., Wallin B. (2016). FRI0209 MORAb-022, an Anti-Granulocyte Macrophage-Colony Stimulating Factor (GM-CSF) Monoclonal Antibody (MAB): RESULTS of the First Study in Patients with Mild-to-Moderate Rheumatoid Arthritis (RA).

[139] Behrens F., Tak P.P., Østergaard M., Stoilov R., Wiland P., Huizinga T.W., Berenfus V.Y., Vladeva S., Rech J., Rubbert-Roth A. (2015). MOR103, a human monoclonal antibody to granulocyte-macrophage colony-stimulating factor, in the treatment of patients with moderate rheumatoid arthritis: Results of a phase Ib/IIa randomised, double-blind, placebo-controlled, dose-escalation trial. Ann. Rheum. Dis..

[140] Burmester G.R., Weinblatt M.E., McInnes I.B., Porter D., Barbarash O., Vatutin M., Szombati I., Esfandiari E., Sleeman M.A., Kane C.D. (2013). Efficacy and safety of mavrilimumab in subjects with rheumatoid arthritis. Ann. Rheum. Dis..

[141] Burmester G.R., McInnes I.B., Kremer J.M., Miranda P., Korkosz M., Vencovsky J., Rubbert-Roth A., Mysler E., Sleeman M.A., Godwood A. (2015). Efficacy and safety of mavrilimumab, A fully human Gm–CSFR-Alpha monoclonal antibody in patients with rheumatoid arthritis: Primary results from the Earth Explorer 1 Study. Ann. Rheum. Dis..

[142] Burmester G.R., McInnes I.B., Kremer J., Miranda P., Korkosz M., Vencovsky J., Rubbert-Roth A., Mysler E., Sleeman M.A., Godwood A. (2017). A randomised phase IIb study of mavrilimumab, a novel GM-CSF receptor alpha monoclonal antibody, in the treatment of rheumatoid arthritis. Ann. Rheum. Dis..

[143] Jin D.C., Wu C.Y., Roskos L.K., Godwood A., Close D., Wang B. (2015). AB0445 Exposure–Efficacy Analysis of Mavrilimumab in Rheumatoid Arthritis: Modeling and Simulation of Phase II Clinical Data. Ann. Rheum. Dis..

[144] Burmester G.R., McInnes I.B., Kremer J.M., Miranda P., Vencovský J., Godwood A., Albulescu M., Michaels M.A., Guo X., Close D. (2018). Mavrilimumab, a Fully Human Granulocyte-Macrophage Colony-Stimulating Factor Receptor α Monoclonal Antibody: Long-Term Safety and Efficacy in Patients with Rheumatoid Arthritis. Arthritis Rheumatol..

[145] Weinblatt M., McInnes I., Kremer J., Miranda P., Vencovský J., Godwood A., Albulescu M., Close D., Burmester G. (2016). Earth explorer 2, a phase IIb exploratory study evaluating efficacy and safety of mavrilimumab, a fully human granulocyte-macrophage colony-stimulating factor receptor-alpha monoclonal antibody, and the tumor necrosis factor antagonist golimumab in rheumatoid arthritis. Ann. Rheum. Dis..

[146] Chen Y., Li F., Hua M., Liang M., Song C. (2023). Role of GM-CSF in lung balance and disease. Front. Immunol..

[147] Lazarus H.M., Pitts K., Wang T., Lee E., Buchbinder E., Dougan M., Armstrong D.G., Paine R., Ragsdale C.E., Boyd T. (2023). Recombinant GM-CSF for diseases of GM-CSF insufficiency: Correcting dysfunctional mononuclear phagocyte disorders. Front. Immunol..

[148] McCormick T.S., Hejal R.B., Leal L.O., Ghannoum M.A. (2022). GM-CSF: Orchestrating the Pulmonary Response to Infection. Front. Pharmacol..

[149] Wishart A.L., Pechacek J., Rosen L.B., Desai J.V., Zarakas M.A., Webb T., Pittaluga S., Seyedmousavi A., Hohl T.M., Kuhns D.B. (2025). Neutralizing GM-CSF autoantibodies impair neutrophil antifungal effector function in a patient with aspergillosis. J. Infect..

[150] Ataya A., Knight V., Carey B.C., Lee E., Tarling E.J., Wang T. (2021). The Role of GM-CSF Autoantibodies in Infection and Autoimmune Pulmonary Alveolar Proteinosis: A Concise Review. Front. Immunol..

[151] Campo I., Carey B.C., Paracchini E., Kadija Z., De Silvestri A., Rodi G., De Amici M., Torre C., Zorzetto M., Griese M. (2024). Inhaled recombinant GM-CSF reduces the need for whole lung lavage and improves gas exchange in autoimmune pulmonary alveolar proteinosis patients. Eur. Respir. J..

[152] Bonfield T.L., Kavuru M.S., Thomassen M.J. (2002). Anti-GM-CSF titer predicts response to GM-CSF therapy in pulmonary alveolar proteinosis. Clin. Immunol..

[153] Campo I., Meloni F., Gahlemann M., Sauter W., Ittrich C., Schoelch C., Trapnell B.C., Gupta A. (2022). An exploratory study investigating biomarkers associated with autoimmune pulmonary alveolar proteinosis (aPAP). Sci. Rep..

[154] Chen W., Feng X., Yao L.K., Li X., Yang Z.M., Qin X.Y., Li Y., Qiu Y. (2025). Exogenous GM-CSF therapy for autoimmune pulmonary alveolar proteinosis: A systematic literature review. Front. Med..

[155] Chen H., Gao N., Fan D., Wu J., Zhu J., Li J., Wang J., Chen Y., An J. (2012). Suppressive effects on the immune response and protective immunity to a JEV DNA vaccine by co-administration of a GM-CSF-expressing plasmid in mice. PLoS ONE.

[156] Parmiani G., Castelli C., Pilla L., Santinami M., Colombo M.P., Rivoltini L. (2007). Opposite immune functions of GM-CSF administered as vaccine adjuvant in cancer patients. Ann. Oncol..

[157] Yu T.W., Chueh H.Y., Tsai C.C., Lin C.T., Qiu J.T. (2016). Novel GM-CSF-based vaccines: One small step in GM-CSF gene optimization, one giant leap for human vaccines. Hum. Vaccin. Immunother..

[158] Henrickson S.E., Ruffner M.A., Kwan M. (2016). Unintended Immunological Consequences of Biologic Therapy. Curr. Allergy Asthma Rep..

[159] Zhang F., Jonsson A.H., Nathan A., Millard N., Curtis M., Xiao Q., Gutierrez-Arcelus M., Apruzzese W., Watts G.F.M., Weisenfeld D. (2023). Deconstruction of rheumatoid arthritis synovium defines inflammatory subtypes. Nature.

[160] Hanlon M.M., Smith C.M., Canavan M., Neto N.G.B., Song Q., Lewis M.J., O’Rourke A.M., Tynan O., Barker B.E., Gallagher P. (2024). Loss of synovial tissue macrophage homeostasis precedes rheumatoid arthritis clinical onset. Sci. Adv..

[161] Achuthan A., Cook A.D., Lee M.C., Saleh R., Khiew H.W., Chang M.W., Louis C., Fleetwood A.J., Lacey D.C., Christensen A.D. (2016). Granulocyte macrophage colony-stimulating factor induces CCL17 production via IRF4 to mediate inflammation. J. Clin. Investig..

[162] Sharma S.D., Bluett J. (2024). Towards Personalized Medicine in Rheumatoid Arthritis. Open Access Rheumatol..

[163] Momoi Y., Kumagai S., Nishikawa H. (2025). Immunogenomic precision medicine: A personalized approach based on immunogenomic cancer evolution. Int. Immunol..

[164] Silva R.C.M.C., Travassos L.H., Dutra F.F. (2024). The dichotomic role of single cytokines: Fine-tuning immune responses. Cytokine.

[165] Chang C.J., Chen Y.H., Huang K.W., Cheng H.W., Chan S.F., Tai K.F., Hwang L.H. (2007). Combined GM-CSF and IL-12 gene therapy synergistically suppresses the growth of orthotopic liver tumors. Hepatology.

[166] Zhu C., Shi Y., Li Q., Luo L., Li X., Luo Z., Lu Y., Zhang J., Jiang M., Qin B. (2022). Rational administration sequencing of immunochemotherapy elicits powerful anti-tumor effect. J. Control Release.

[167] Kwon O.C., Lee E.J., Chang E.J., Youn J., Ghang B., Hong S., Lee C.K., Yoo B., Kim Y.G. (2018). IL-17A^+^GM-CSF^+^ Neutrophils Are the Major Infiltrating Cells in Interstitial Lung Disease in an Autoimmune Arthritis Model. Front. Immunol..

[168] O’Rielly D.D., Rahman P. (2010). Pharmacogenetics of rheumatoid arthritis: Potential targets from susceptibility genes and present therapies. Pharmgenom. Pers. Med..

[169] Gent Y.Y., Voskuyl A.E., Kloet R.W., van Schaardenburg D., Hoekstra O.S., Dijkmans B.A., Lammertsma A.A., van der Laken C.J. (2012). Macrophage positron emission tomography imaging as a biomarker for preclinical rheumatoid arthritis: Findings of a prospective pilot study. Arthritis Rheum..

[170] Bruijnen S.T.G., Verweij N.J.F., Gent Y.Y.J., Huisman M.C., Windhorst A.D., Kassiou M., van de Ven P.M., Lammertsma A.A., Hoekstra O.S., Voskuyl A.E. (2019). Imaging disease activity of rheumatoid arthritis by macrophage targeting using second generation translocator protein positron emission tomography tracers. PLoS ONE.

[171] Curtis J.R., van der Helm-van Mil A.H., Knevel R., Huizinga T.W., Haney D.J., Shen Y., Ramanujan S., Cavet G., Centola M., Hesterberg L.K. (2012). Validation of a novel multibiomarker test to assess rheumatoid arthritis disease activity. Arthritis Care Res..

[172] Syversen S.W., Landewe R., van der Heijde D., Bathon J.M., Boers M., Bykerk V.P., Fitzgerald O., Gladman D.D., Garnero P., Geusens P. (2009). Testing of the OMERACT 8 draft validation criteria for a soluble biomarker reflecting structural damage in rheumatoid arthritis: A systematic literature search on 5 candidate biomarkers. J. Rheumatol..

[173] Piper C., Pesenacker A.M., Bending D., Thirugnanabalan B., Varsani H., Wedderburn L.R., Nistala K. (2014). T cell expression of granulocyte-macrophage colony-stimulating factor in juvenile arthritis is contingent upon Th17 plasticity. Arthritis Rheumatol..

[174] Malengier-Devlies B., Bernaerts E., Ahmadzadeh K., Filtjens J., Vandenhaute J., Boeckx B., Burton O., De Visscher A., Mitera T., Berghmans N. (2022). Role for Granulocyte Colony-Stimulating Factor in Neutrophilic Extramedullary Myelopoiesis in a Murine Model of Systemic Juvenile Idiopathic Arthritis. Arthritis Rheumatol..

